# Supplementary figures and images for: Purinergic adipocyte-macrophage crosstalk promotes degeneration of thermogenic brown adipose tissue
Source: EMBO Rep. 2025 Nov 19;26(24):6460–93. doi: 10.1038/s44319-025-00642-y (PMC12715258; doi:10.1038/s44319-025-00642-y)

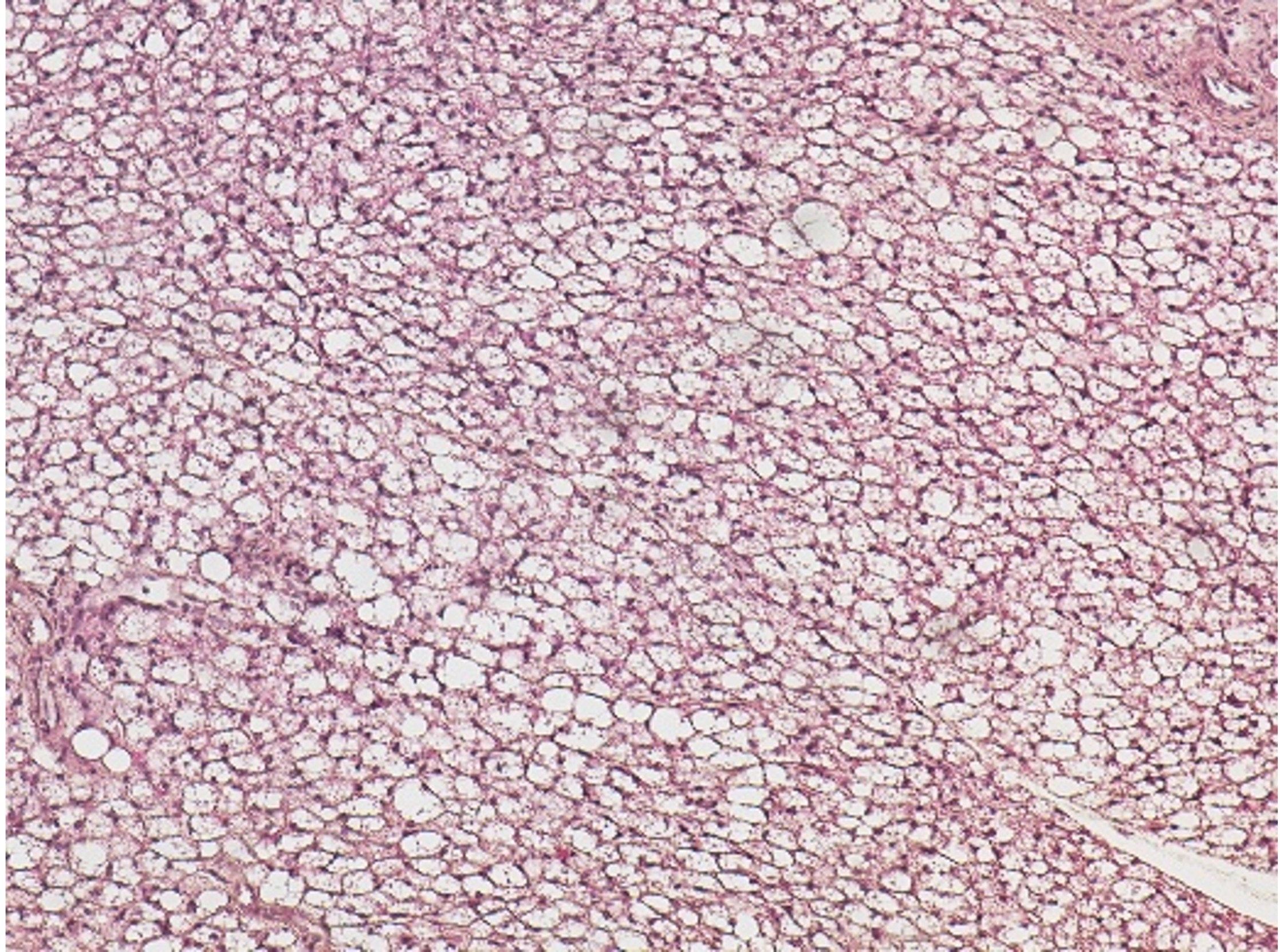

Supplement: Supplementary file 6 — Source data Fig. 1 [file 44319_2025_642_MOESM6_ESM.zip › Figure 1/1A/UCP1KO HE.jpg]

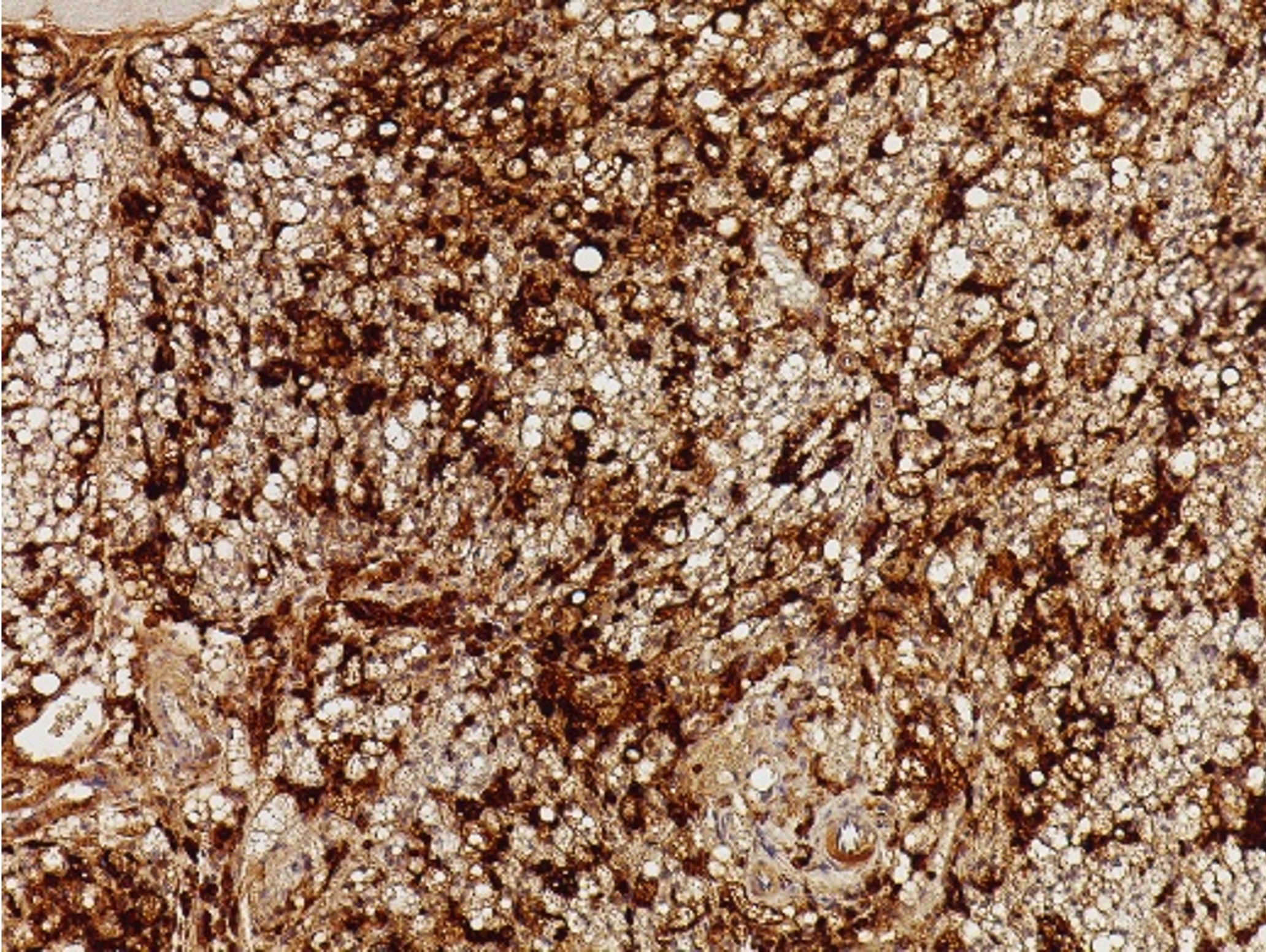

Supplement: Supplementary file 6 — Source data Fig. 1 [file 44319_2025_642_MOESM6_ESM.zip › Figure 1/1A/UCP1KO MAC2.jpg]

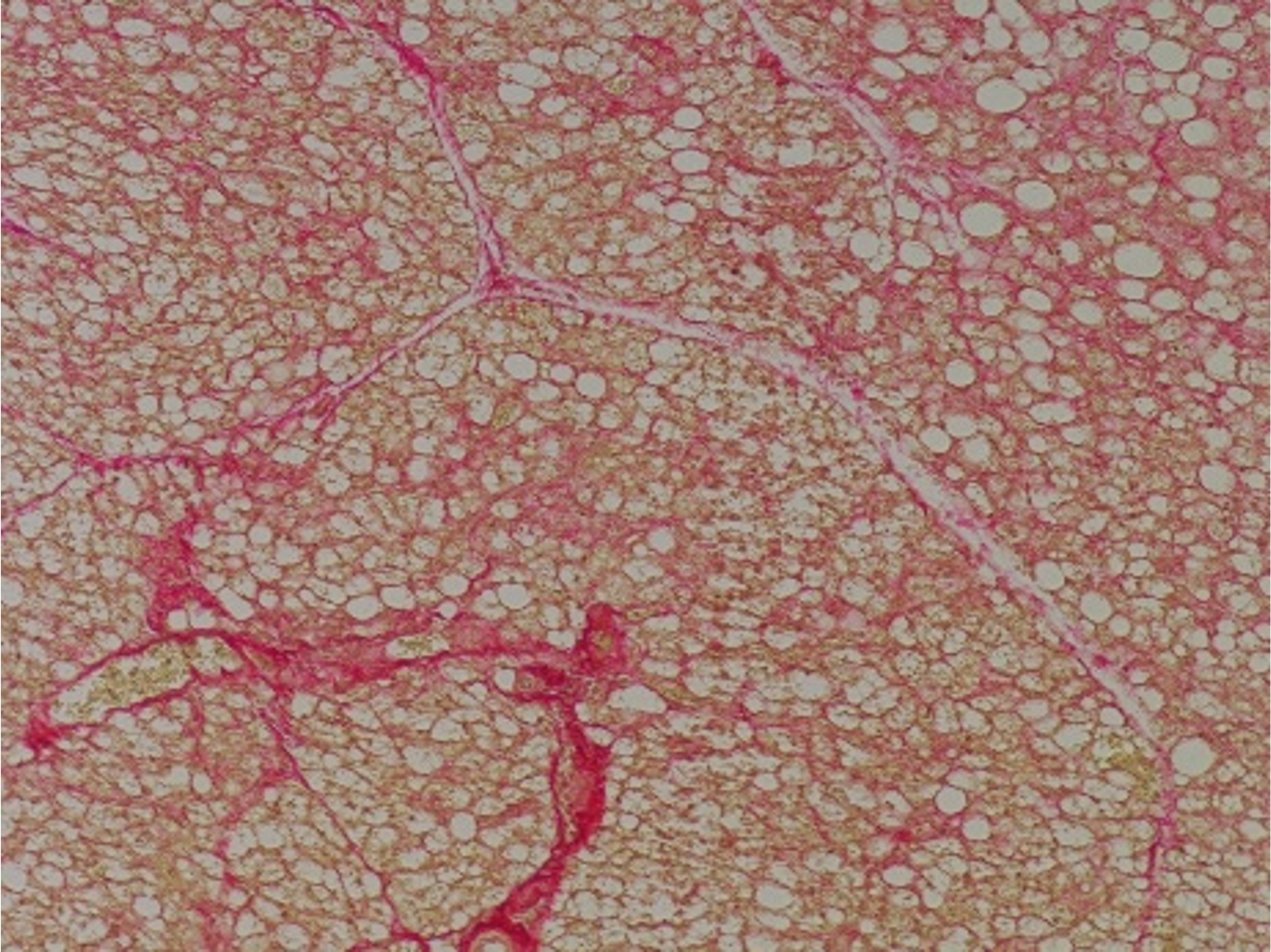

Supplement: Supplementary file 6 — Source data Fig. 1 [file 44319_2025_642_MOESM6_ESM.zip › Figure 1/1A/UCP1KO Sirius Red.jpg]

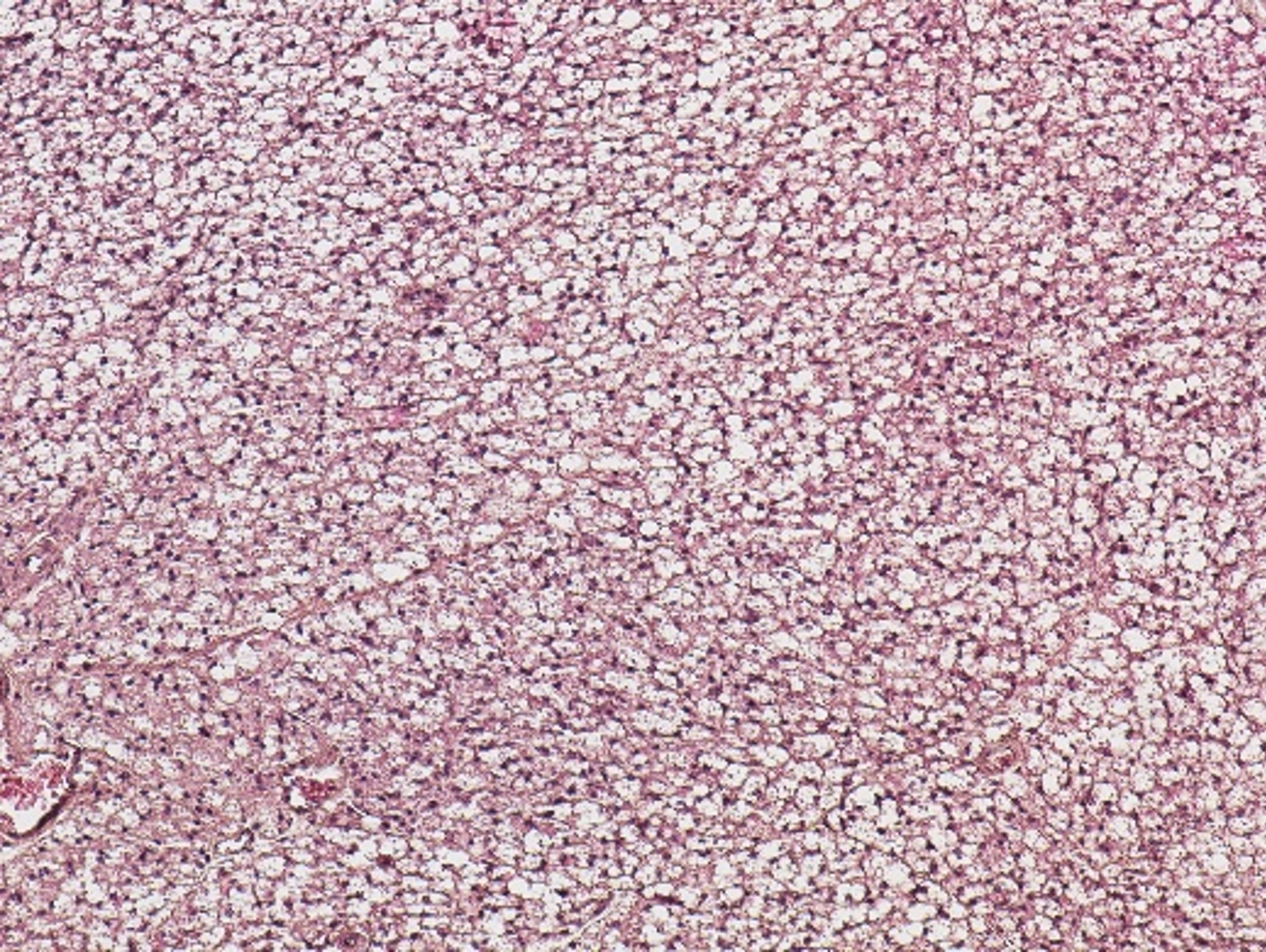

Supplement: Supplementary file 6 — Source data Fig. 1 [file 44319_2025_642_MOESM6_ESM.zip › Figure 1/1A/WT HE.jpg]

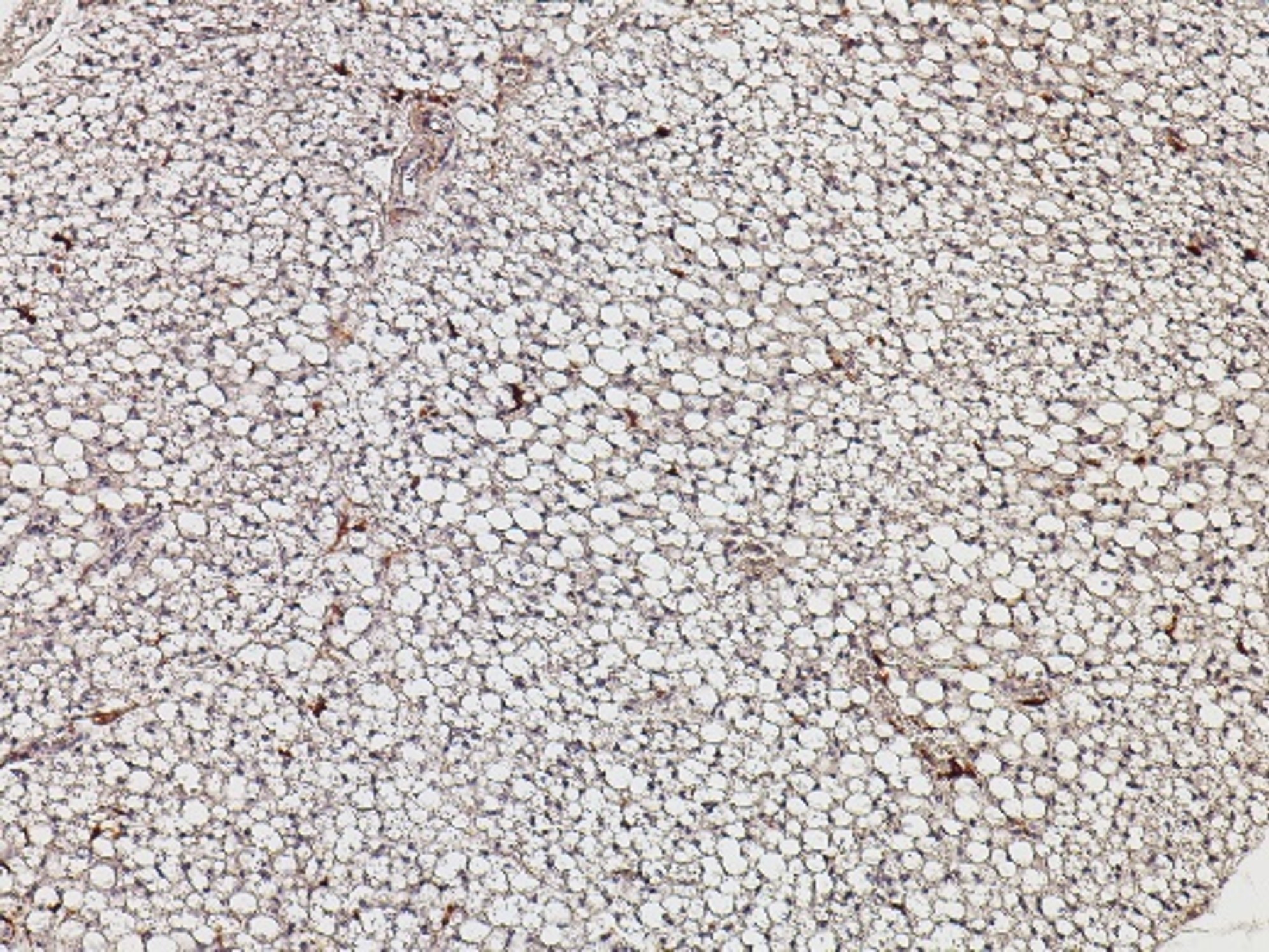

Supplement: Supplementary file 6 — Source data Fig. 1 [file 44319_2025_642_MOESM6_ESM.zip › Figure 1/1A/WT MAC2.jpg]

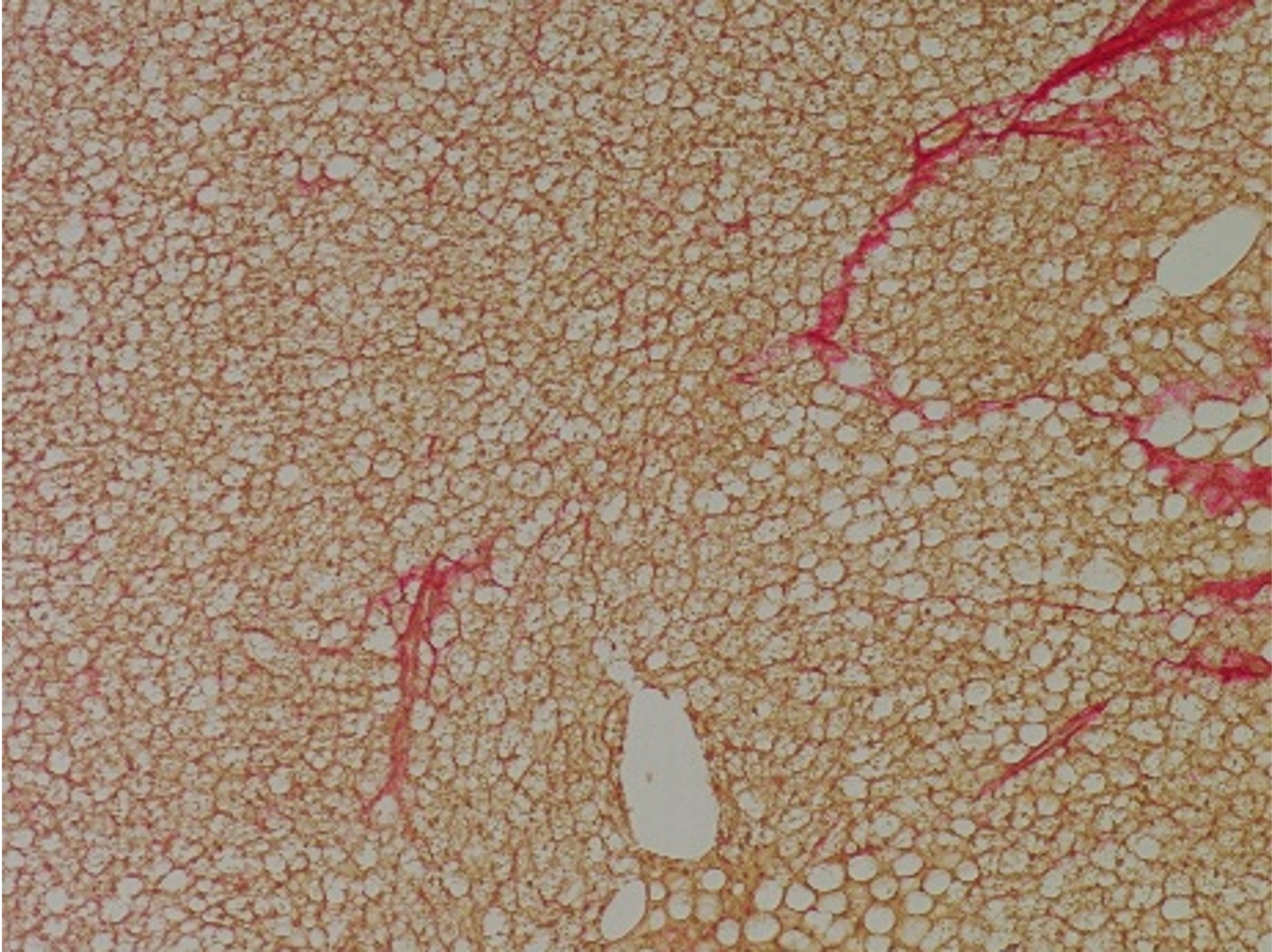

Supplement: Supplementary file 6 — Source data Fig. 1 [file 44319_2025_642_MOESM6_ESM.zip › Figure 1/1A/WT Sirius Red.jpg]

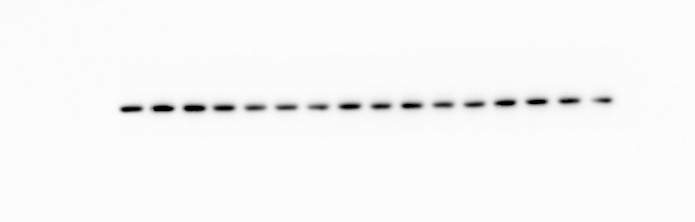

Supplement: Supplementary file 6 — Source data Fig. 1 [file 44319_2025_642_MOESM6_ESM.zip › Figure 1/1C/AKT.jpg]

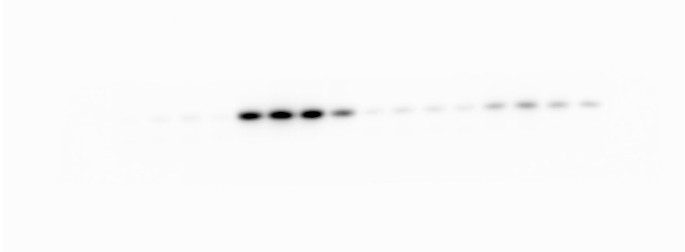

Supplement: Supplementary file 6 — Source data Fig. 1 [file 44319_2025_642_MOESM6_ESM.zip › Figure 1/1C/MAC2.jpg]

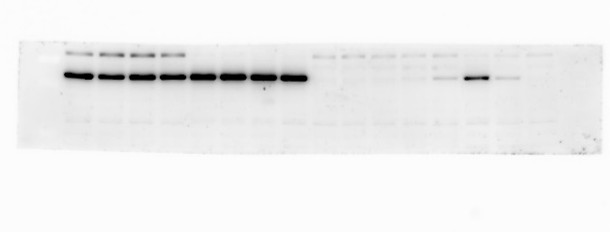

Supplement: Supplementary file 6 — Source data Fig. 1 [file 44319_2025_642_MOESM6_ESM.zip › Figure 1/1C/TH.jpg]

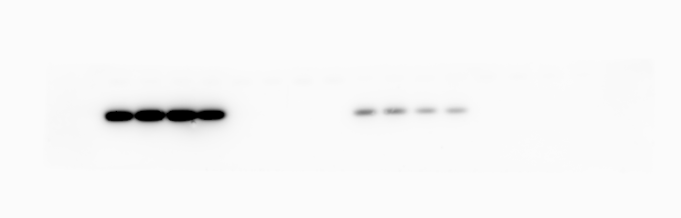

Supplement: Supplementary file 6 — Source data Fig. 1 [file 44319_2025_642_MOESM6_ESM.zip › Figure 1/1C/UCP1.tif]

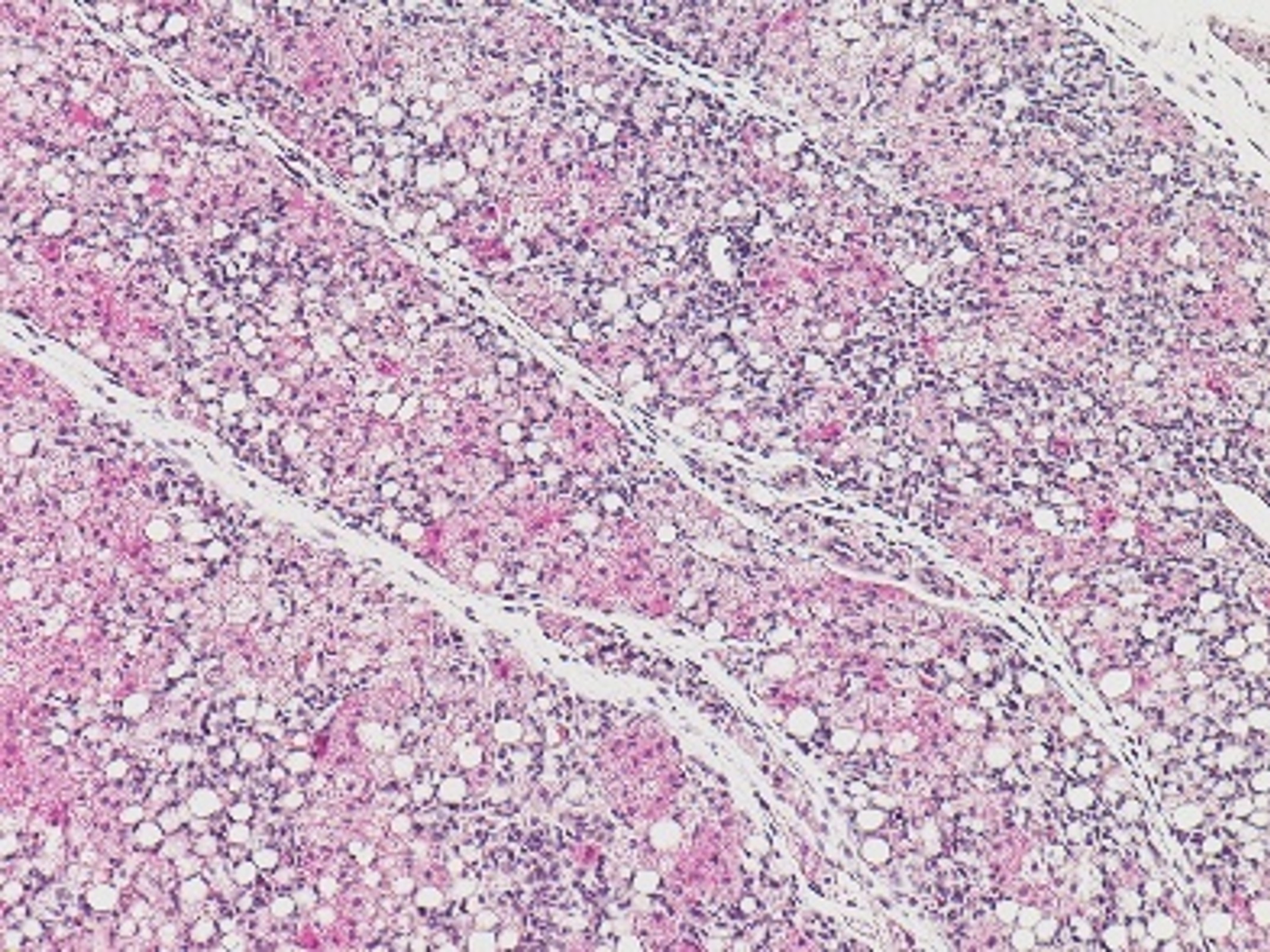

Supplement: Supplementary file 6 — Source data Fig. 1 [file 44319_2025_642_MOESM6_ESM.zip › Figure 1/1F/EtoCL HE.jpg]

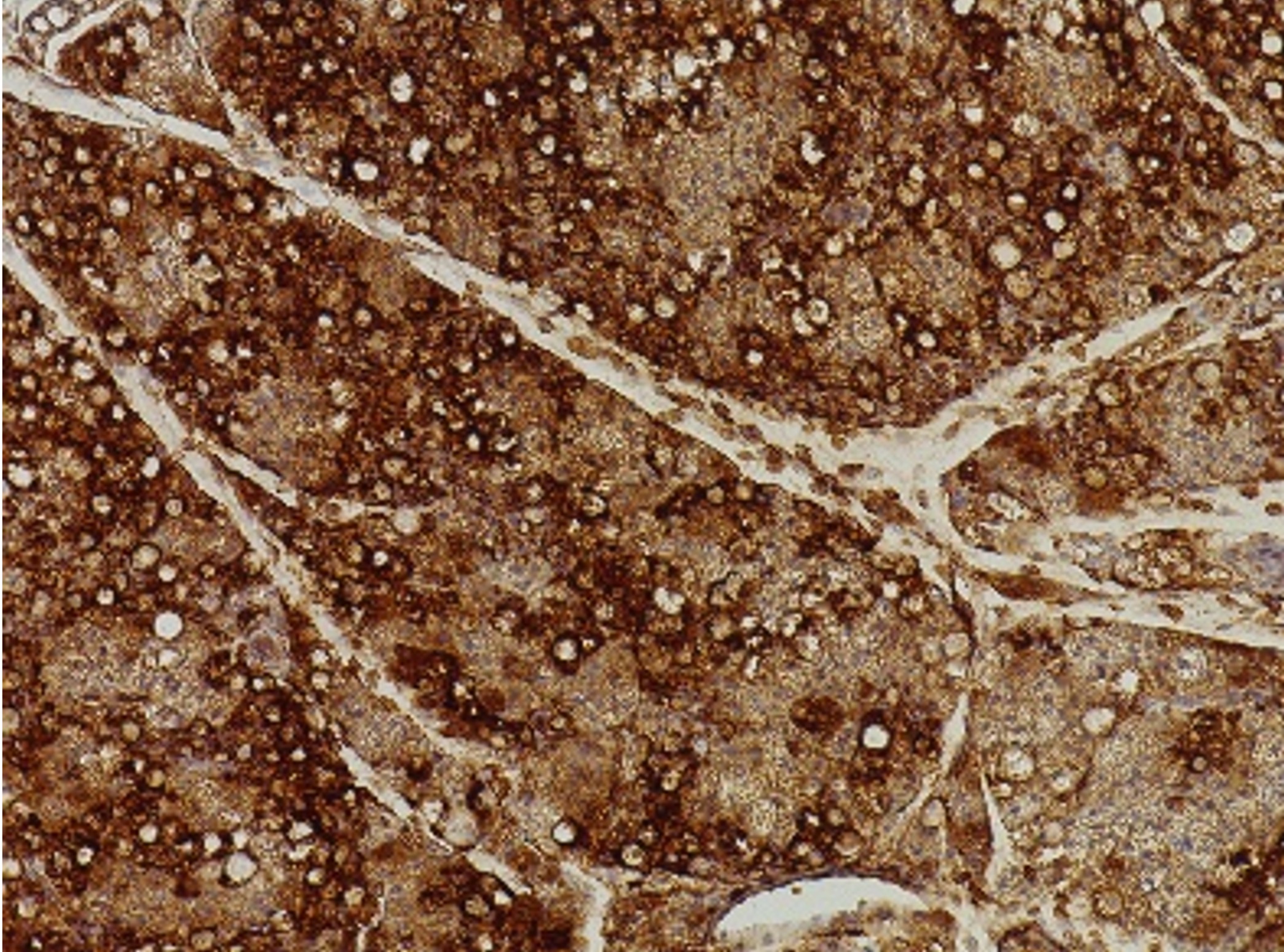

Supplement: Supplementary file 6 — Source data Fig. 1 [file 44319_2025_642_MOESM6_ESM.zip › Figure 1/1F/EtoCL MAC2.jpg]

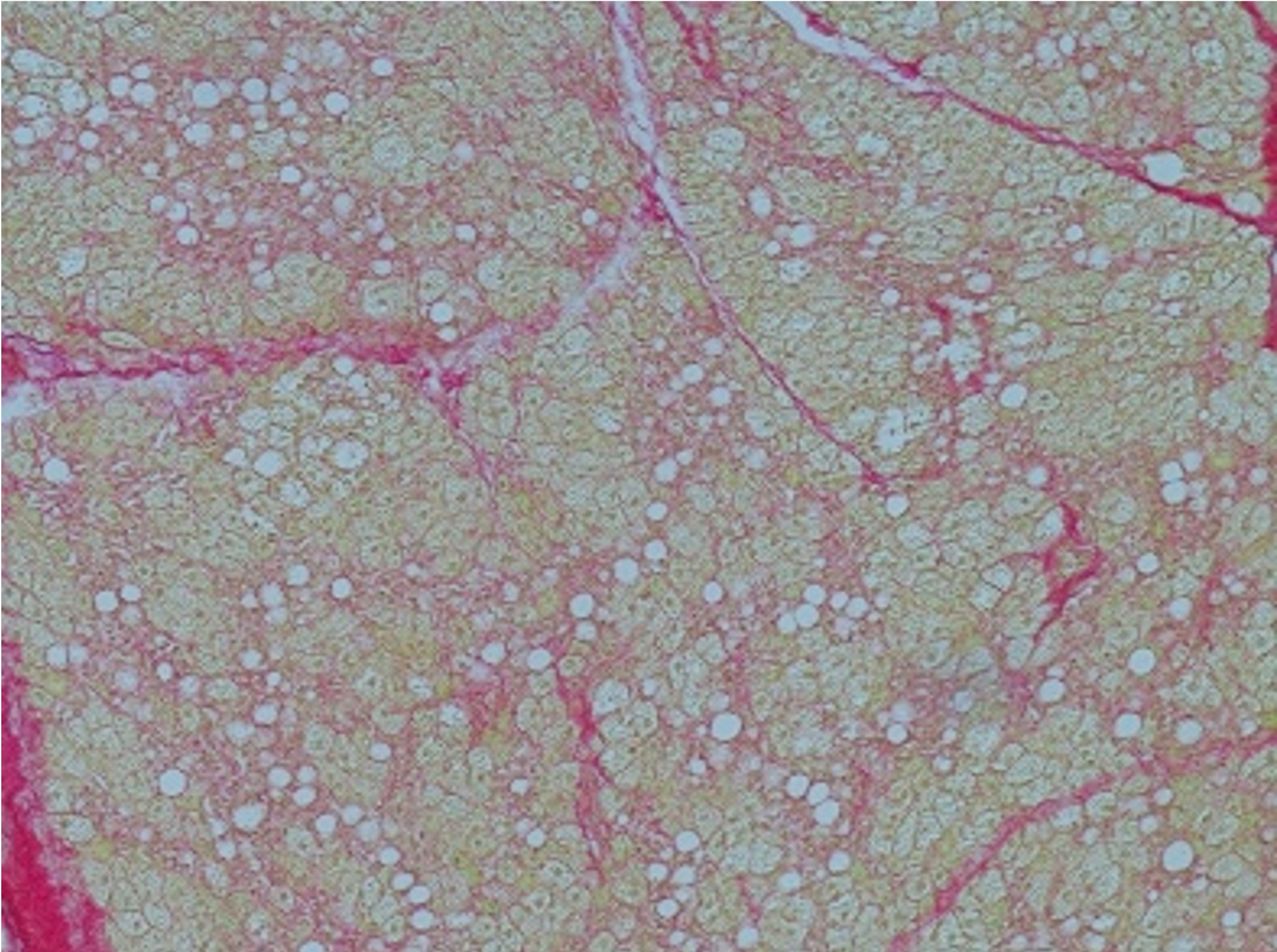

Supplement: Supplementary file 6 — Source data Fig. 1 [file 44319_2025_642_MOESM6_ESM.zip › Figure 1/1F/EtoCL Sirius Red.jpg]

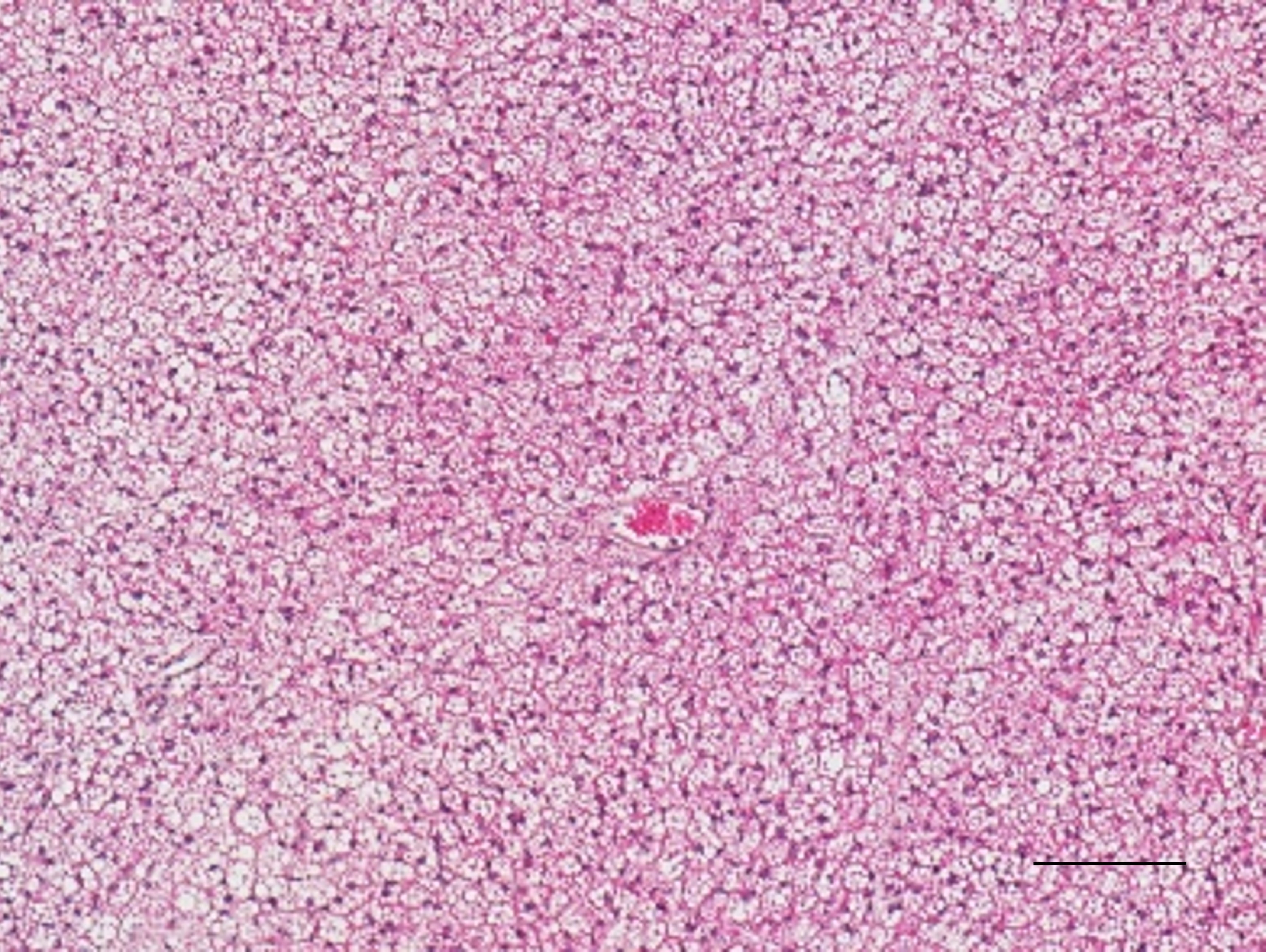

Supplement: Supplementary file 6 — Source data Fig. 1 [file 44319_2025_642_MOESM6_ESM.zip › Figure 1/1F/mockmock HE.jpg]

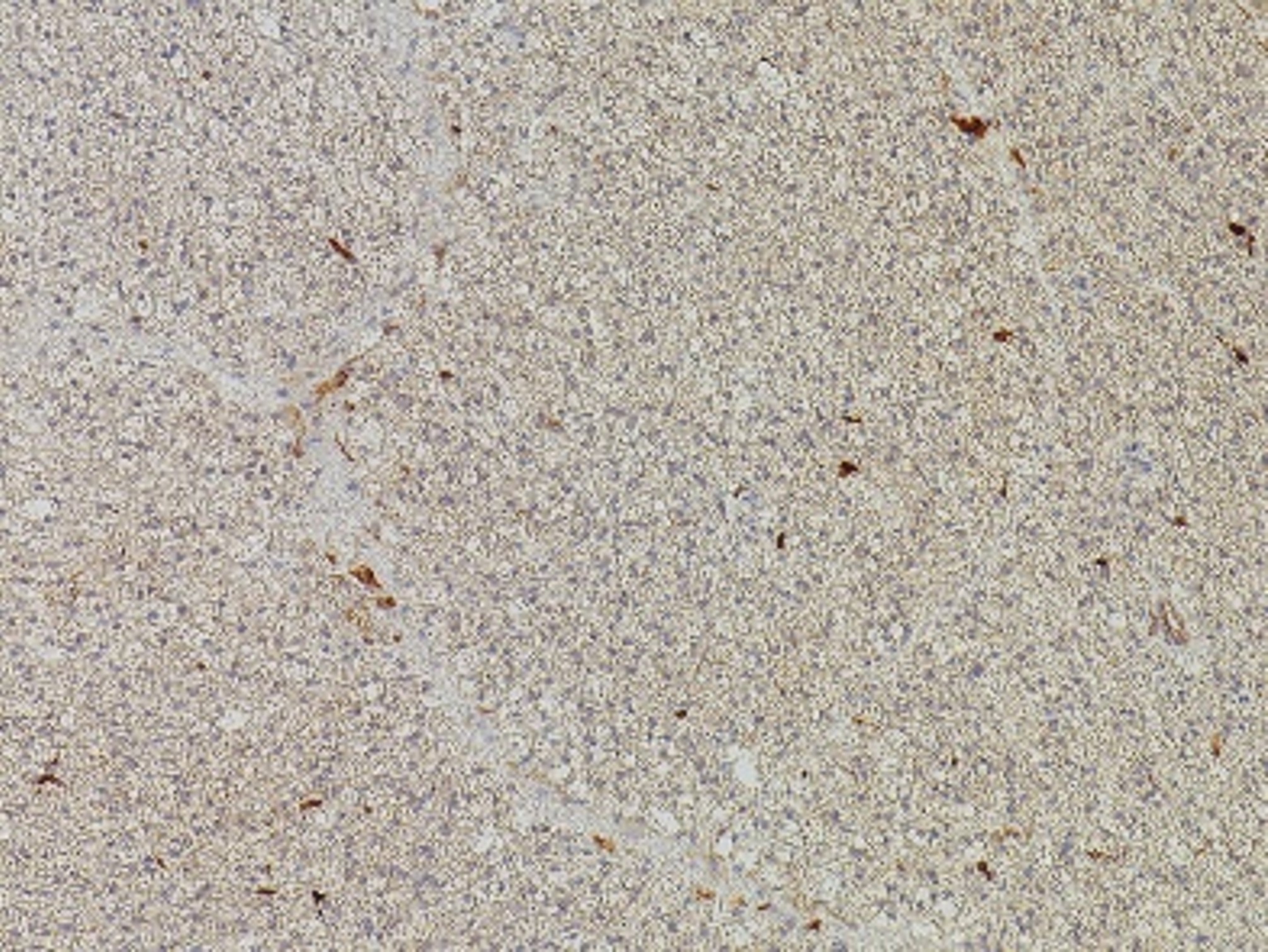

Supplement: Supplementary file 6 — Source data Fig. 1 [file 44319_2025_642_MOESM6_ESM.zip › Figure 1/1F/mockmock MAC2.jpg]

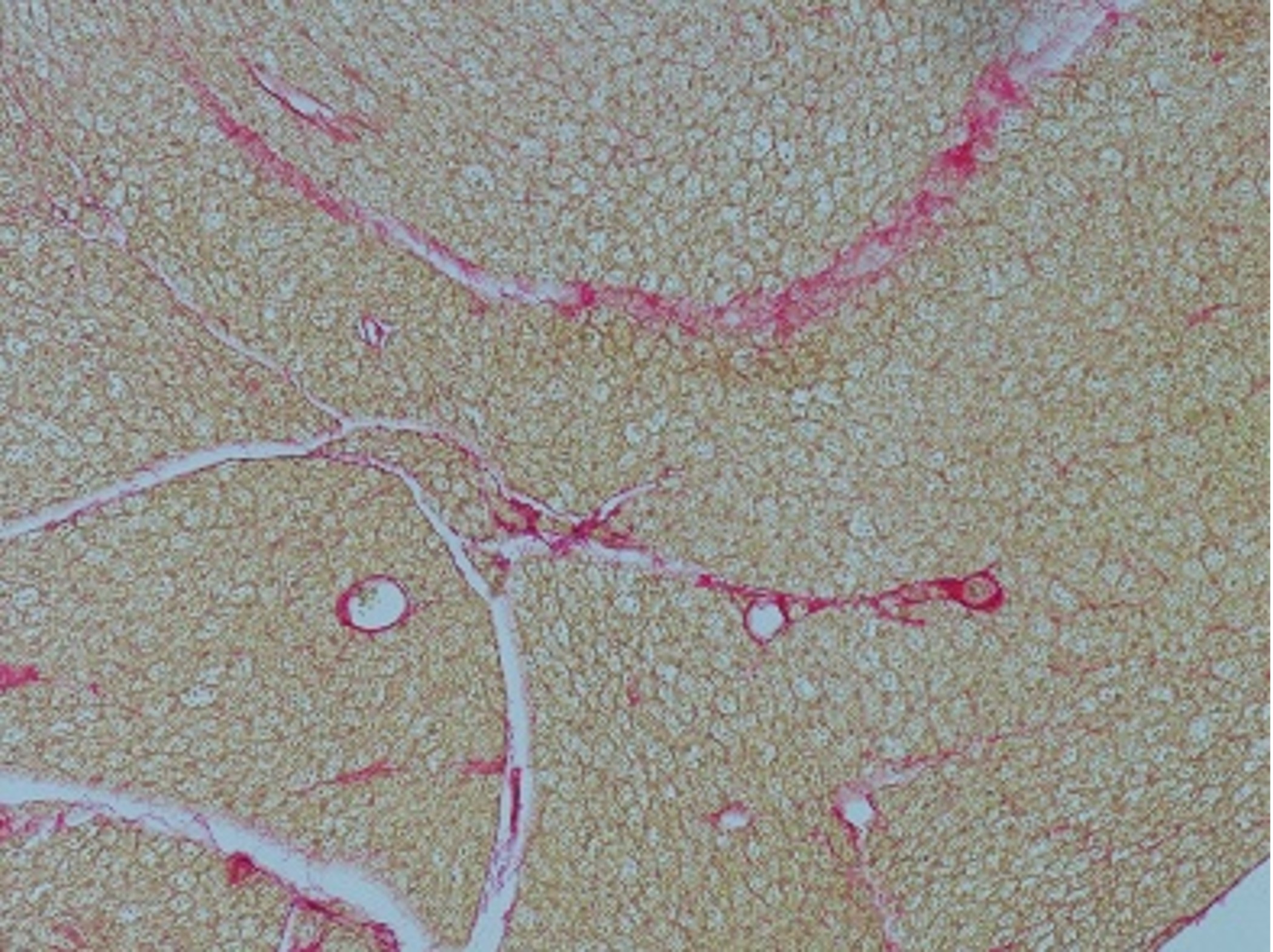

Supplement: Supplementary file 6 — Source data Fig. 1 [file 44319_2025_642_MOESM6_ESM.zip › Figure 1/1F/mockmock Sirius Red.jpg]

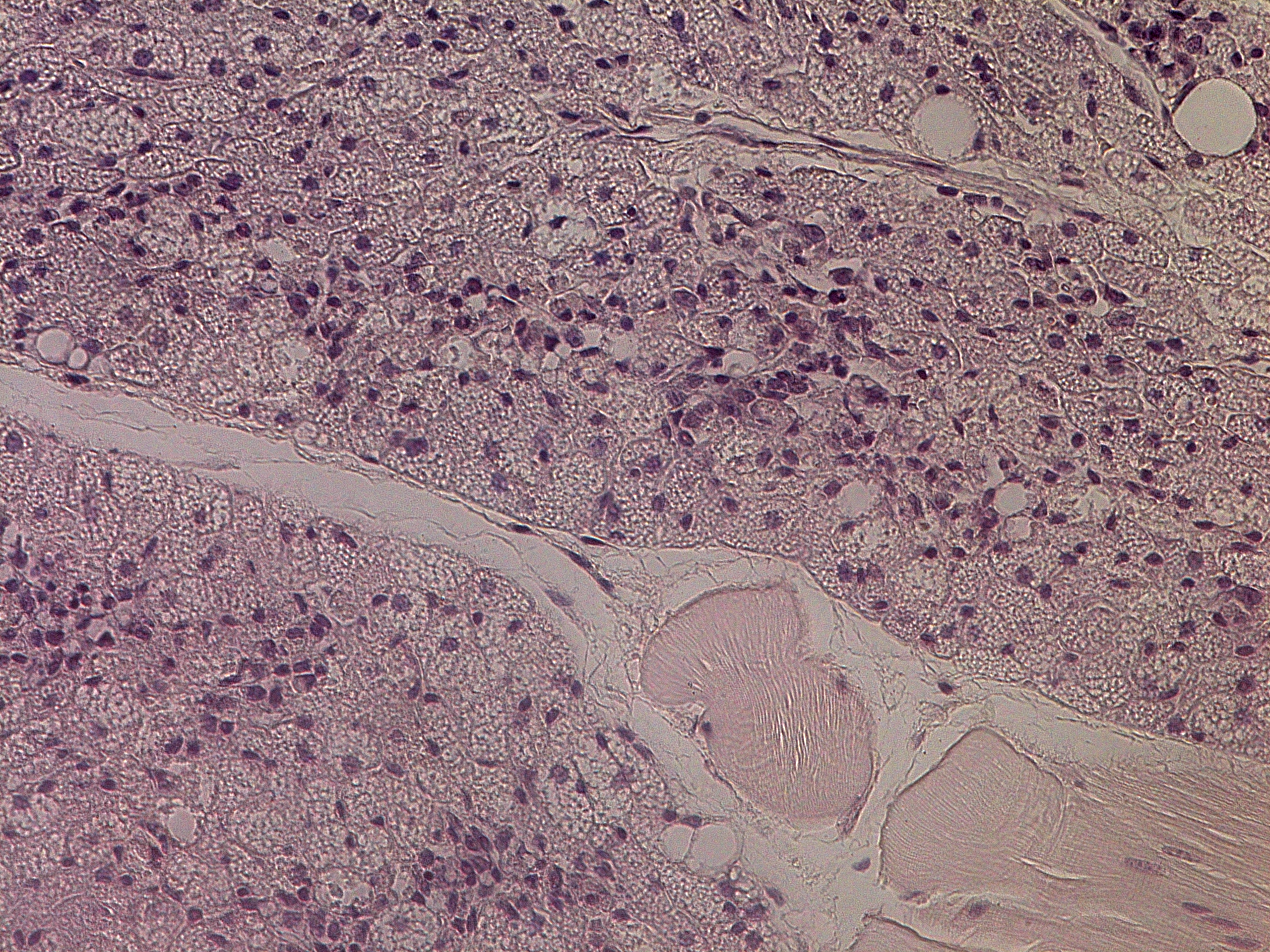

Supplement: Supplementary file 9 — Source data Fig. 4 [file 44319_2025_642_MOESM9_ESM.zip › Figure 4/4A/P2X4KO + control HE.tif]

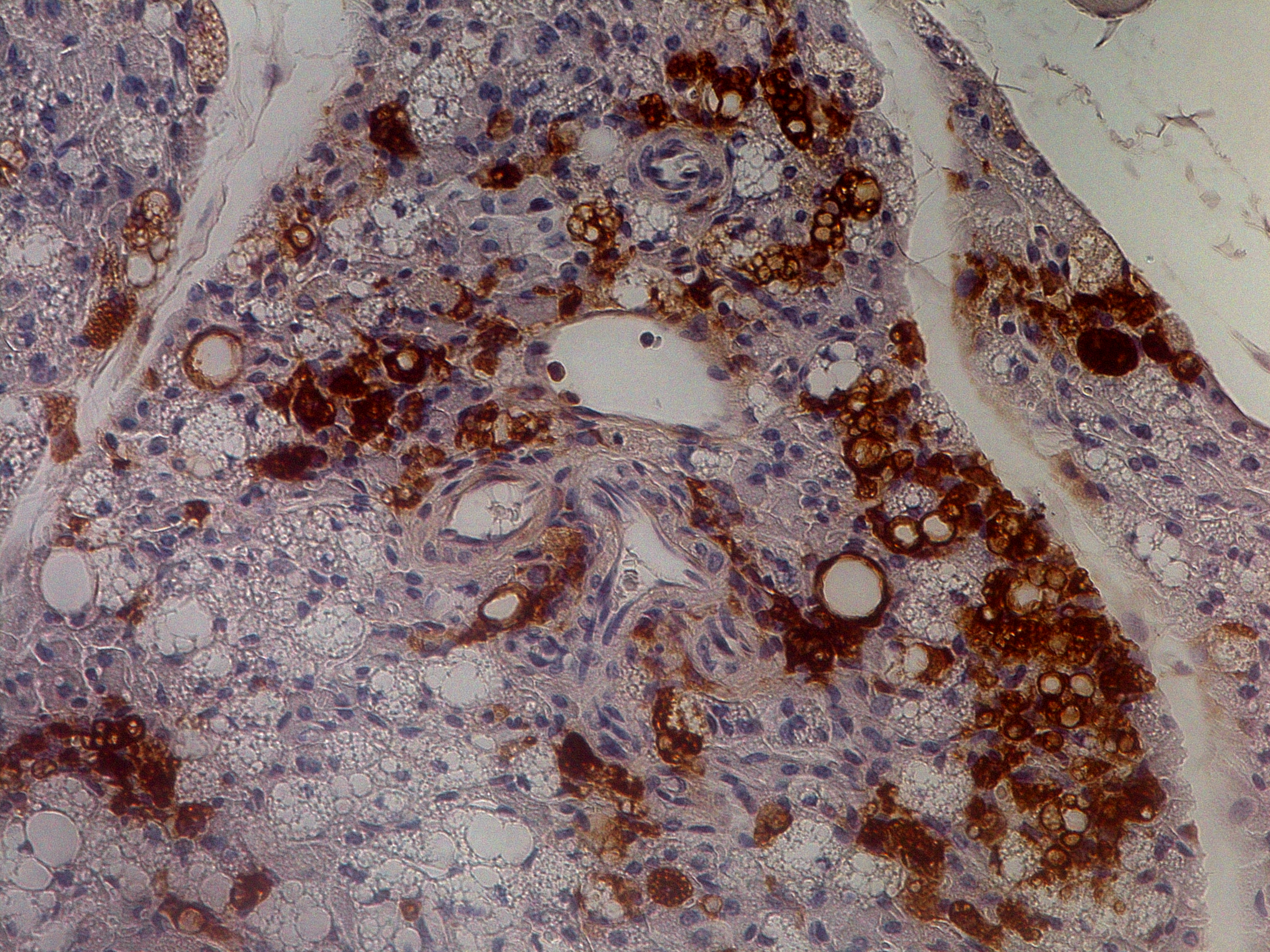

Supplement: Supplementary file 9 — Source data Fig. 4 [file 44319_2025_642_MOESM9_ESM.zip › Figure 4/4A/P2X4KO + control MAC2.tif]

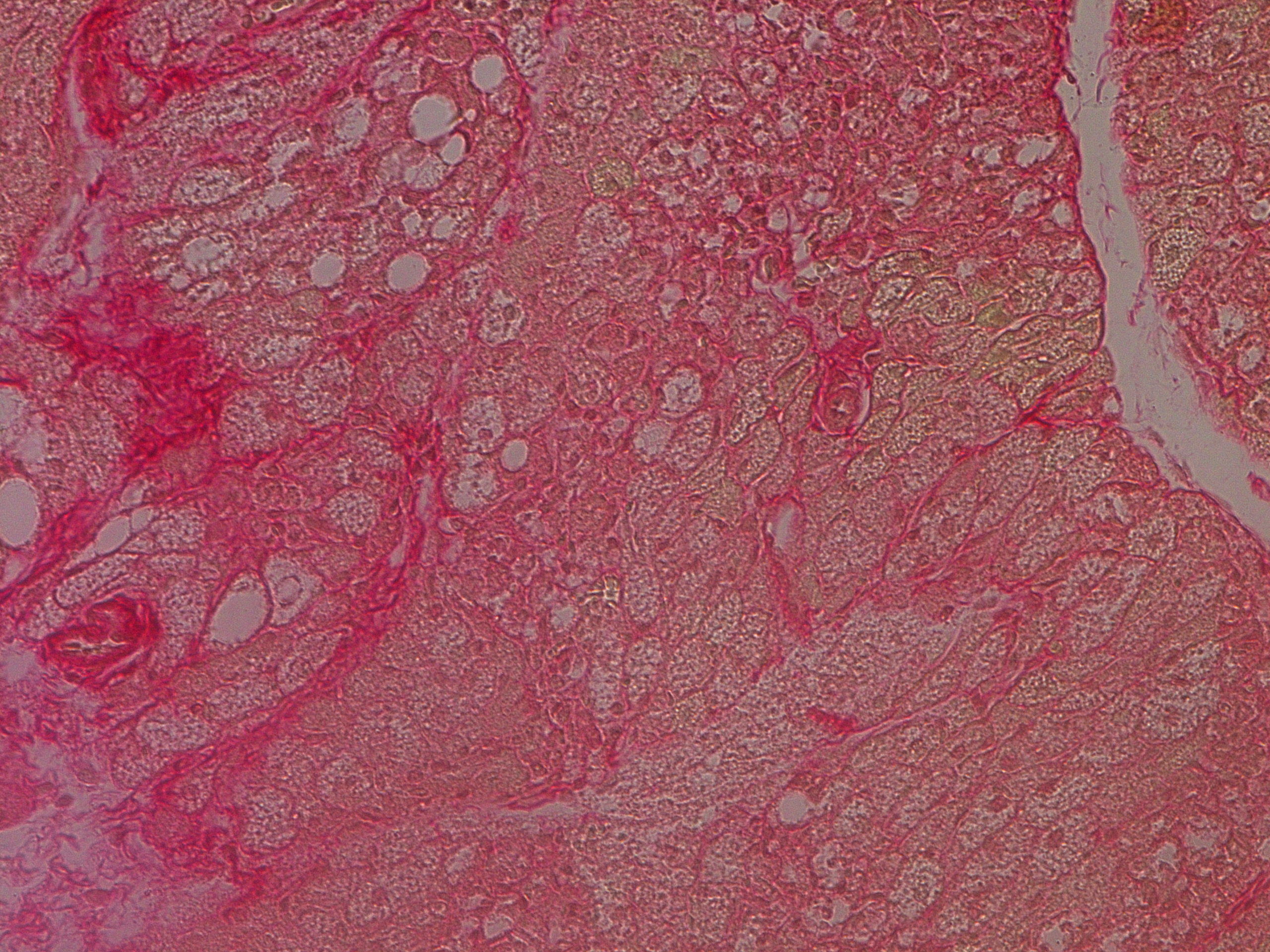

Supplement: Supplementary file 9 — Source data Fig. 4 [file 44319_2025_642_MOESM9_ESM.zip › Figure 4/4A/P2X4KO + control Sirius red.tif]

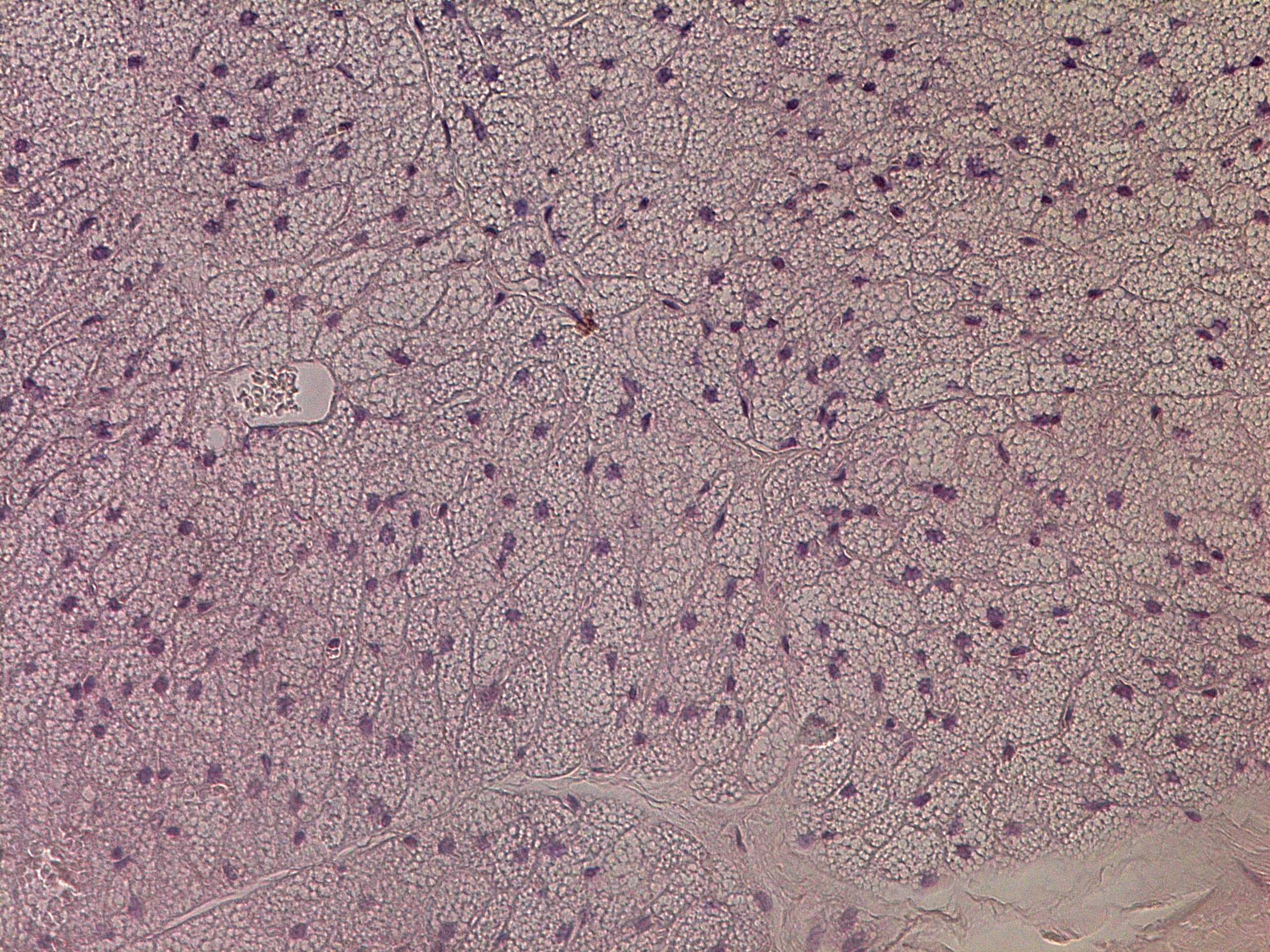

Supplement: Supplementary file 9 — Source data Fig. 4 [file 44319_2025_642_MOESM9_ESM.zip › Figure 4/4A/P2X4KO + P2X7NB HE.tif]

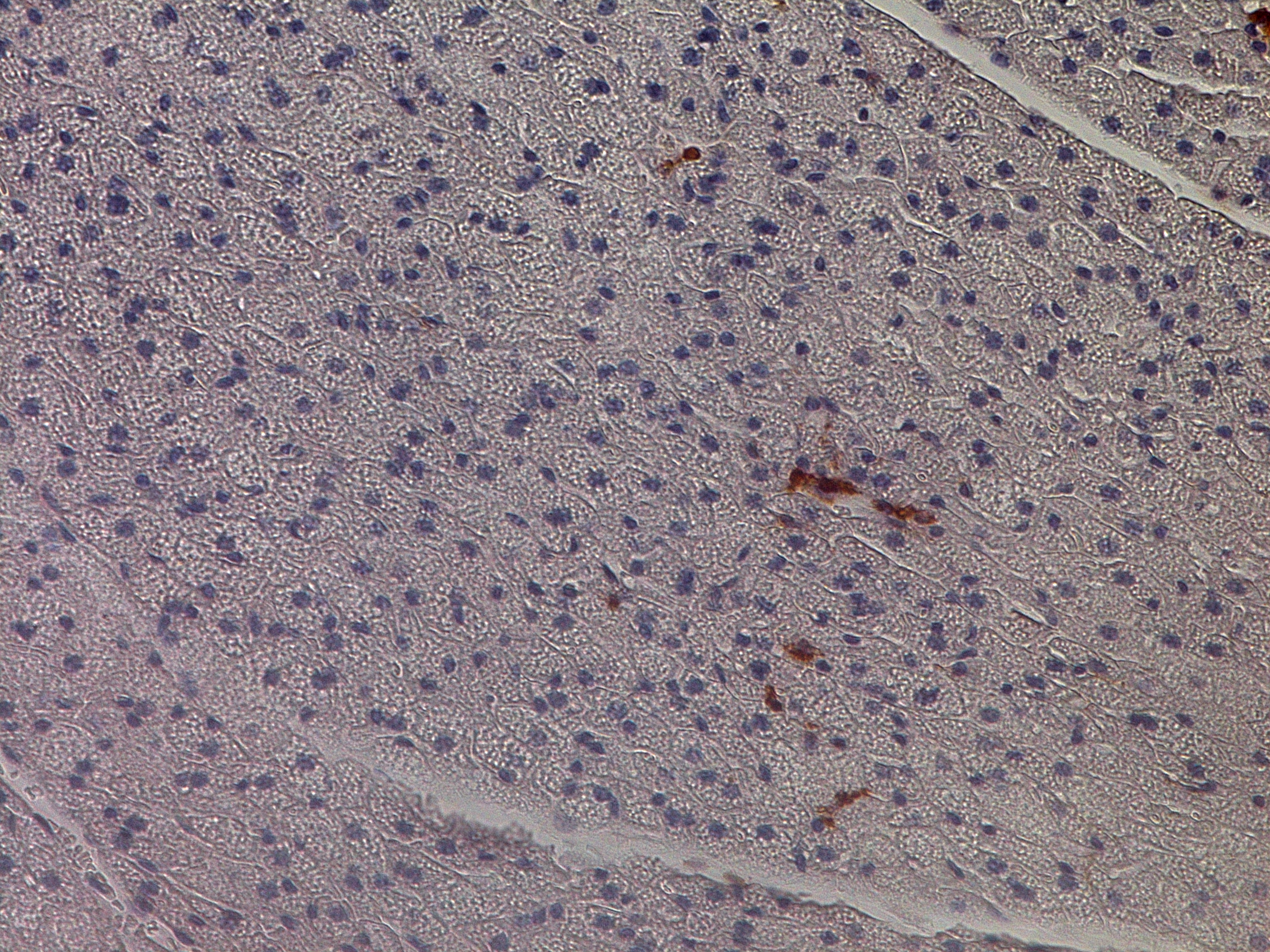

Supplement: Supplementary file 9 — Source data Fig. 4 [file 44319_2025_642_MOESM9_ESM.zip › Figure 4/4A/P2X4KO + P2X7NB MAC2.tif]

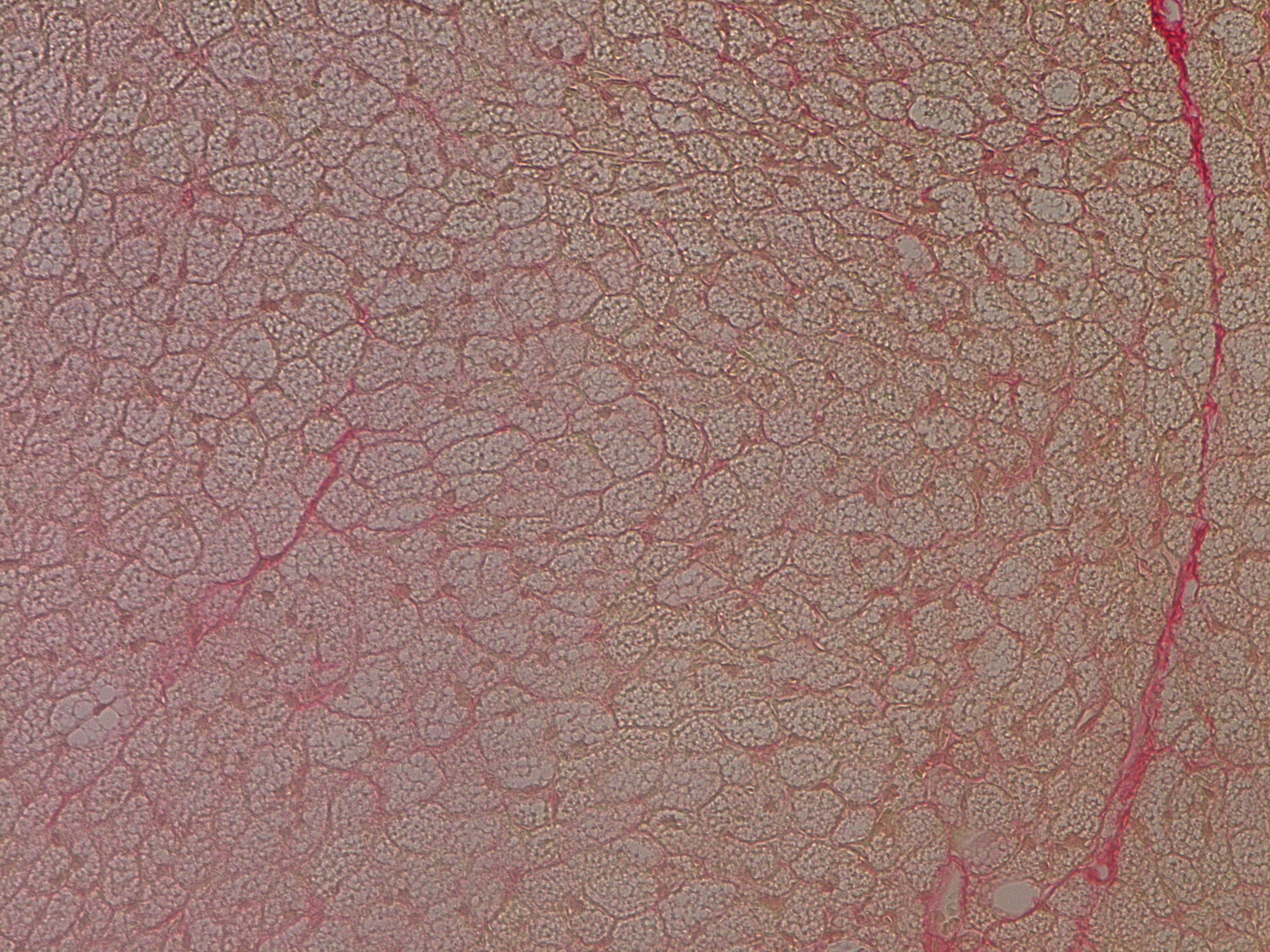

Supplement: Supplementary file 9 — Source data Fig. 4 [file 44319_2025_642_MOESM9_ESM.zip › Figure 4/4A/P2X4KO + P2X7NB Sirius red.tif]

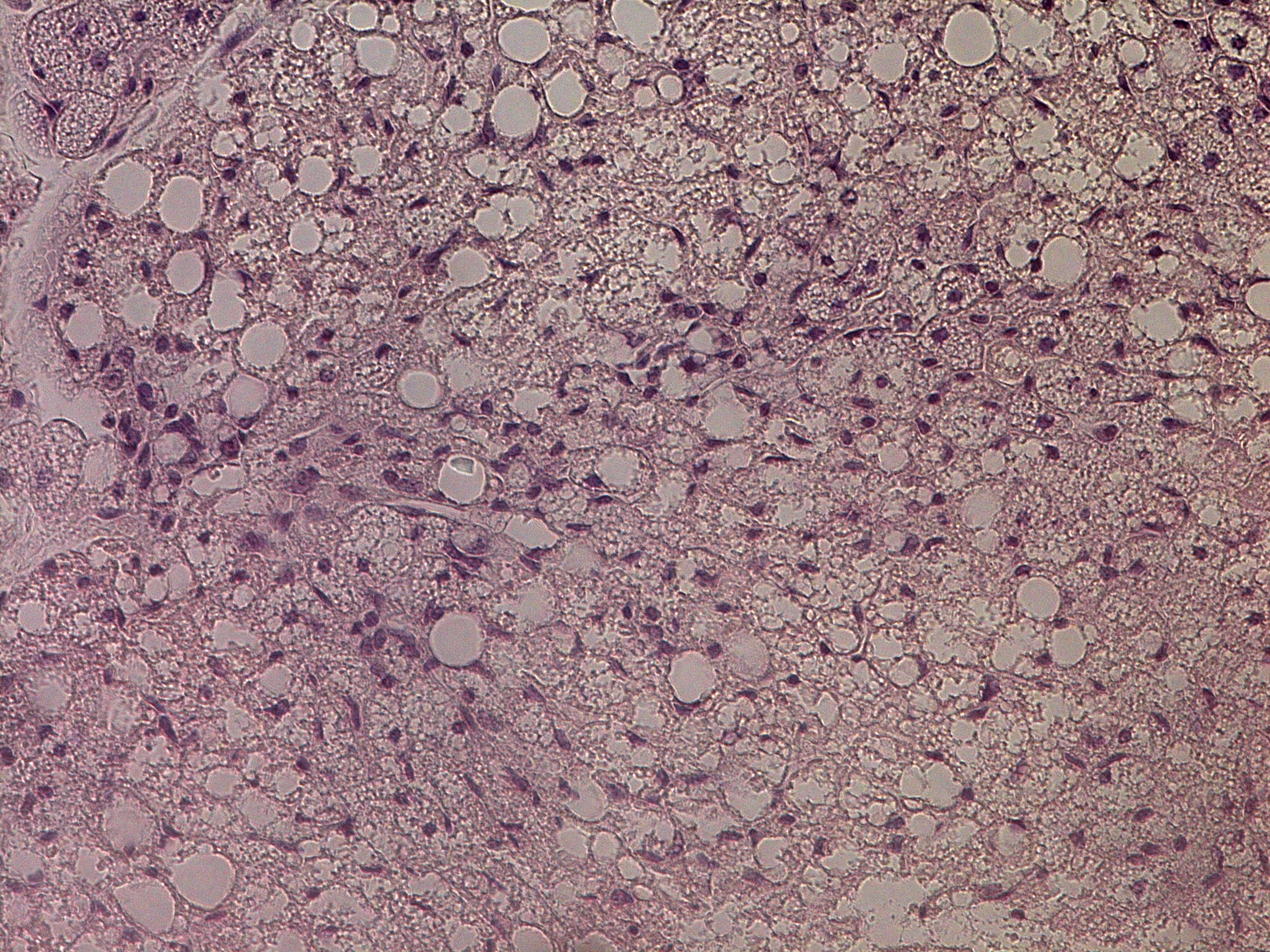

Supplement: Supplementary file 9 — Source data Fig. 4 [file 44319_2025_642_MOESM9_ESM.zip › Figure 4/4A/WT + control HE.tif]

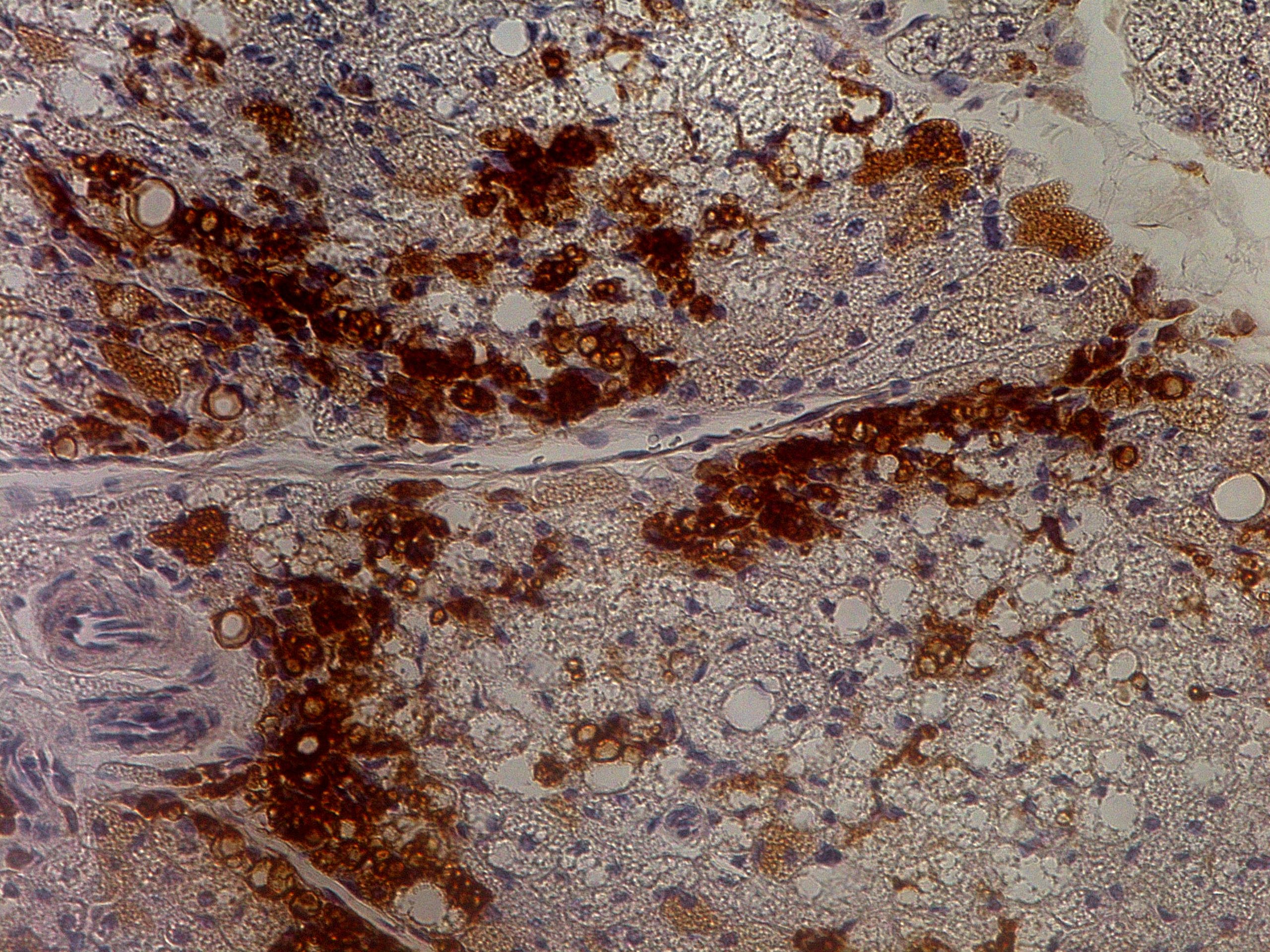

Supplement: Supplementary file 9 — Source data Fig. 4 [file 44319_2025_642_MOESM9_ESM.zip › Figure 4/4A/WT + control MAC2.tif]

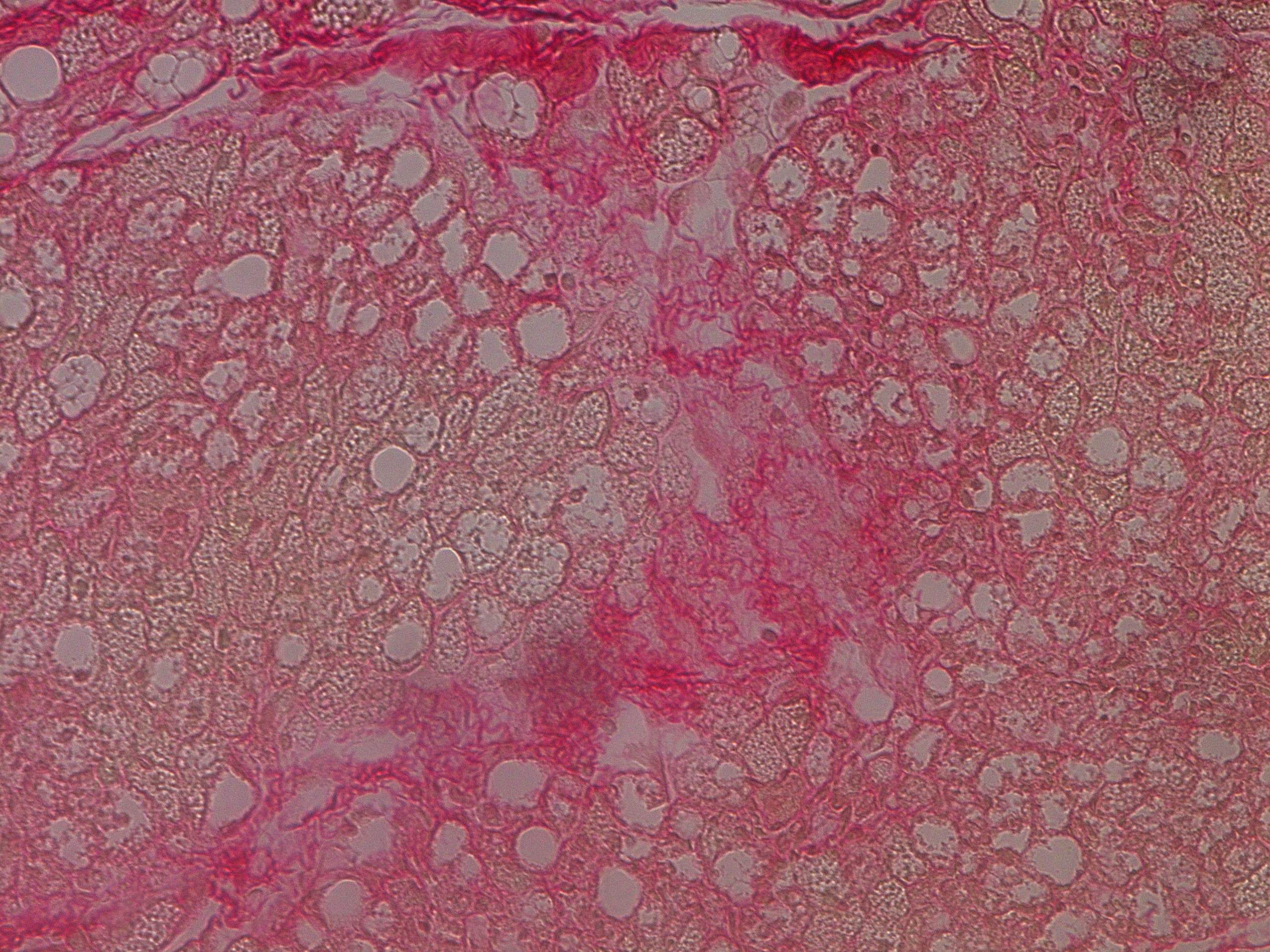

Supplement: Supplementary file 9 — Source data Fig. 4 [file 44319_2025_642_MOESM9_ESM.zip › Figure 4/4A/WT + mock Sirius red.tif]

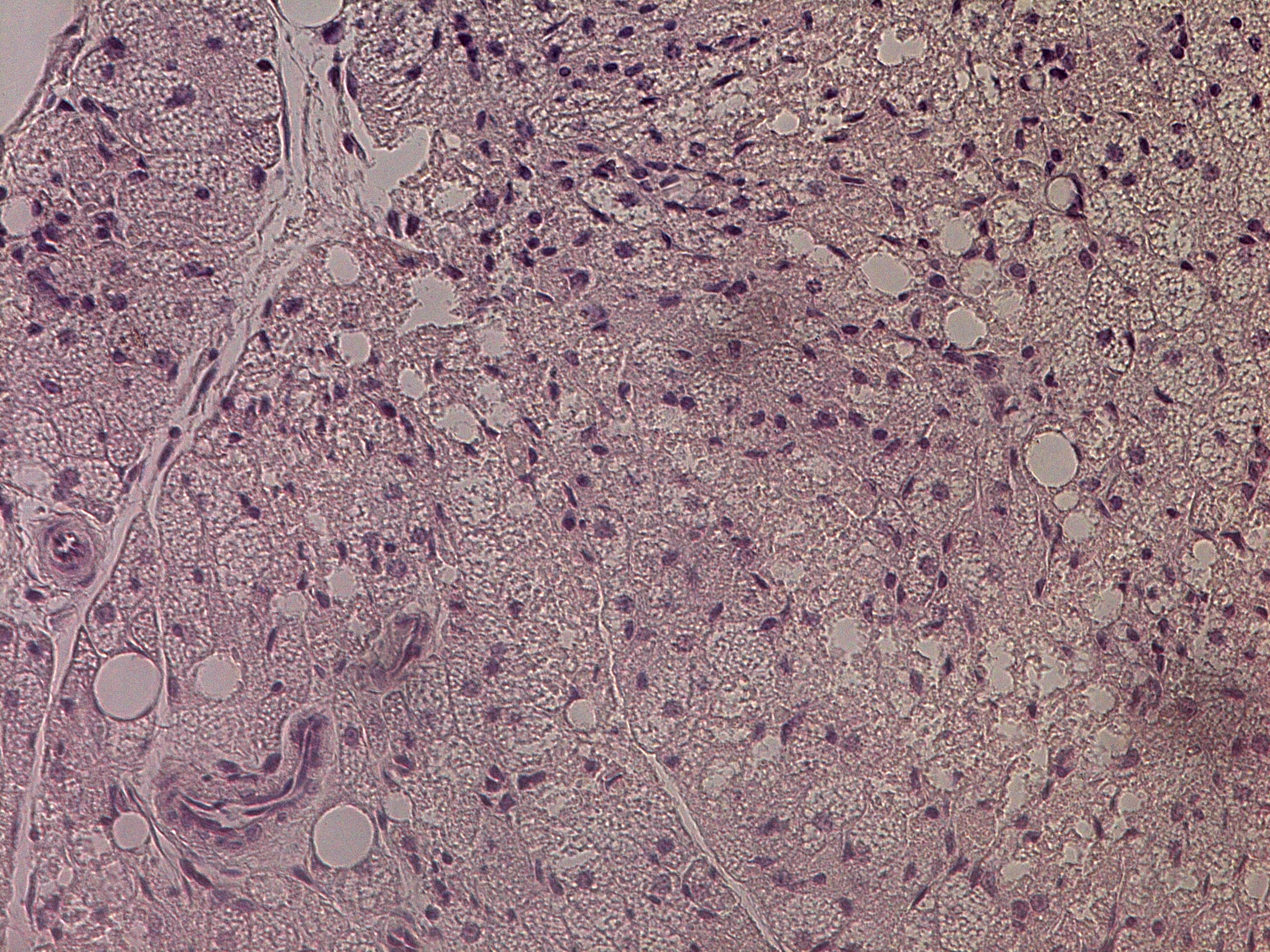

Supplement: Supplementary file 9 — Source data Fig. 4 [file 44319_2025_642_MOESM9_ESM.zip › Figure 4/4A/WT + P2X7NB HE.tif]

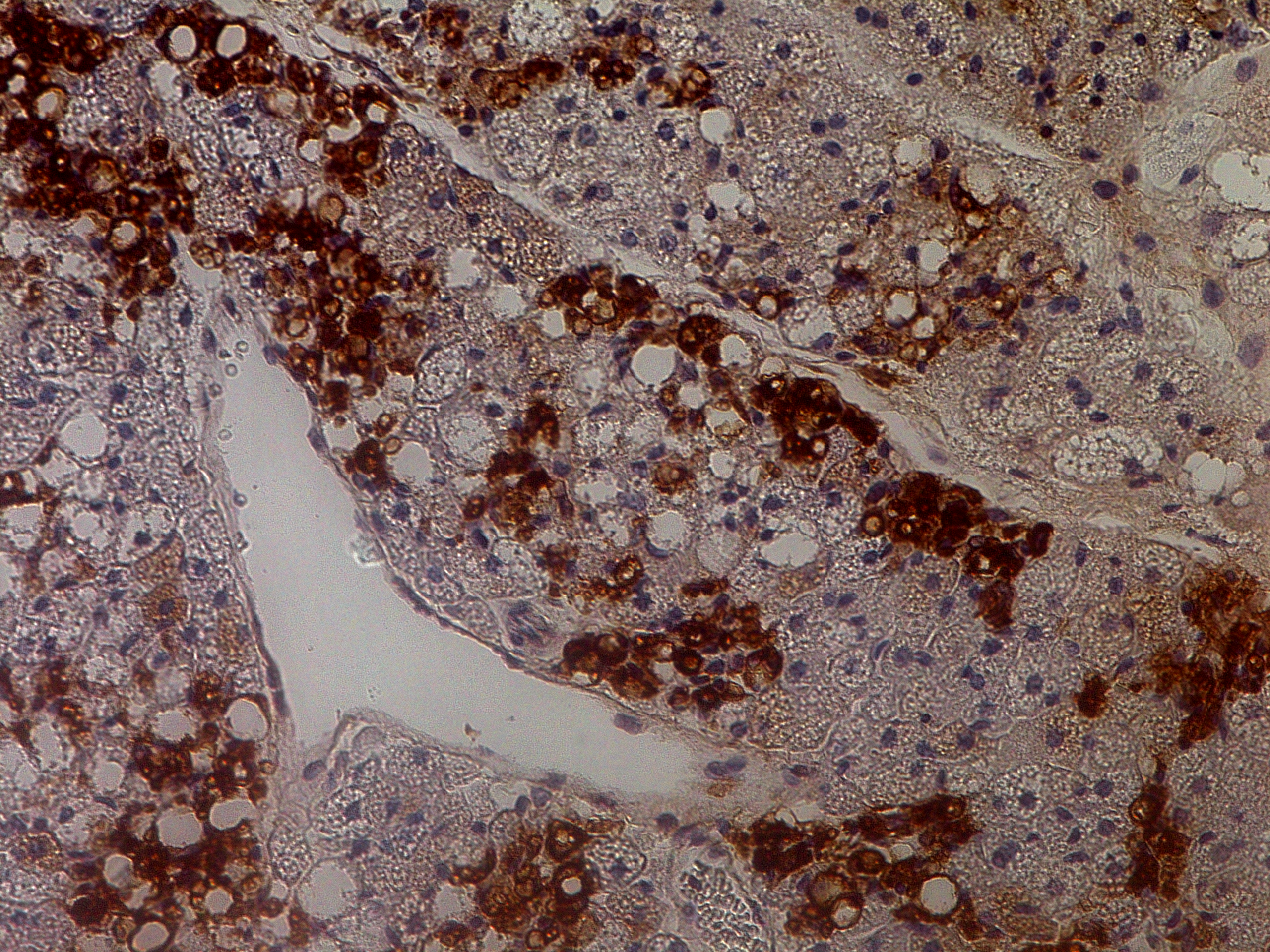

Supplement: Supplementary file 9 — Source data Fig. 4 [file 44319_2025_642_MOESM9_ESM.zip › Figure 4/4A/WT + P2X7NB MAC2.tif]

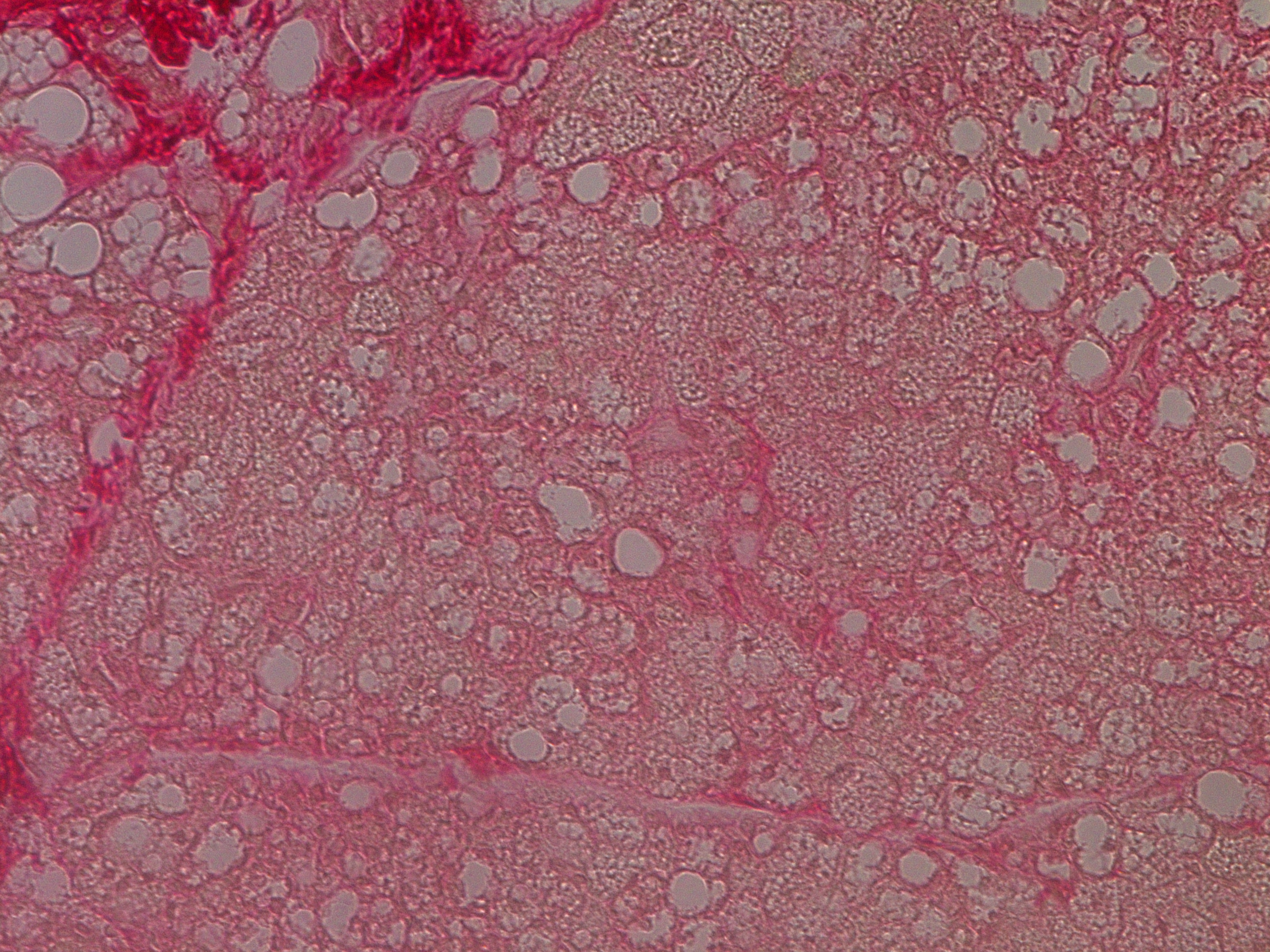

Supplement: Supplementary file 9 — Source data Fig. 4 [file 44319_2025_642_MOESM9_ESM.zip › Figure 4/4A/WT + P2X7NB Sirius red.tif]

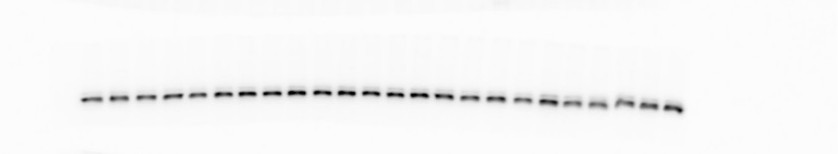

Supplement: Supplementary file 9 — Source data Fig. 4 [file 44319_2025_642_MOESM9_ESM.zip › Figure 4/4C/g-Tubulin.jpg]

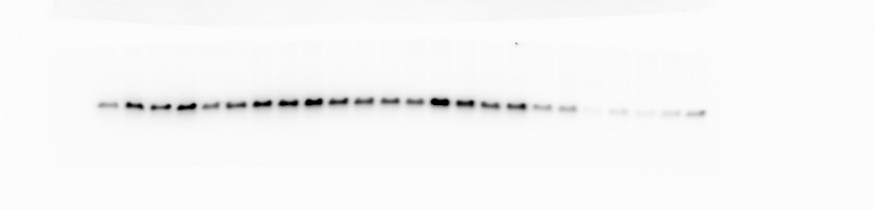

Supplement: Supplementary file 9 — Source data Fig. 4 [file 44319_2025_642_MOESM9_ESM.zip › Figure 4/4C/MAC2.jpg]

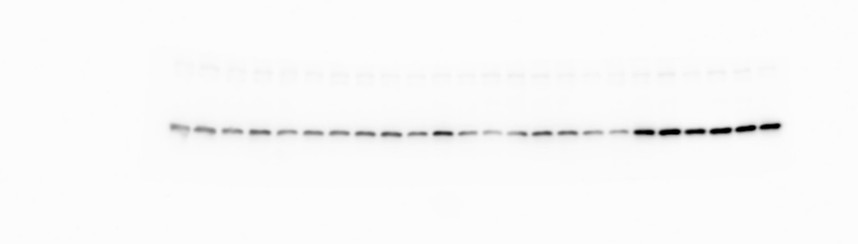

Supplement: Supplementary file 9 — Source data Fig. 4 [file 44319_2025_642_MOESM9_ESM.zip › Figure 4/4C/UCP1.jpg]

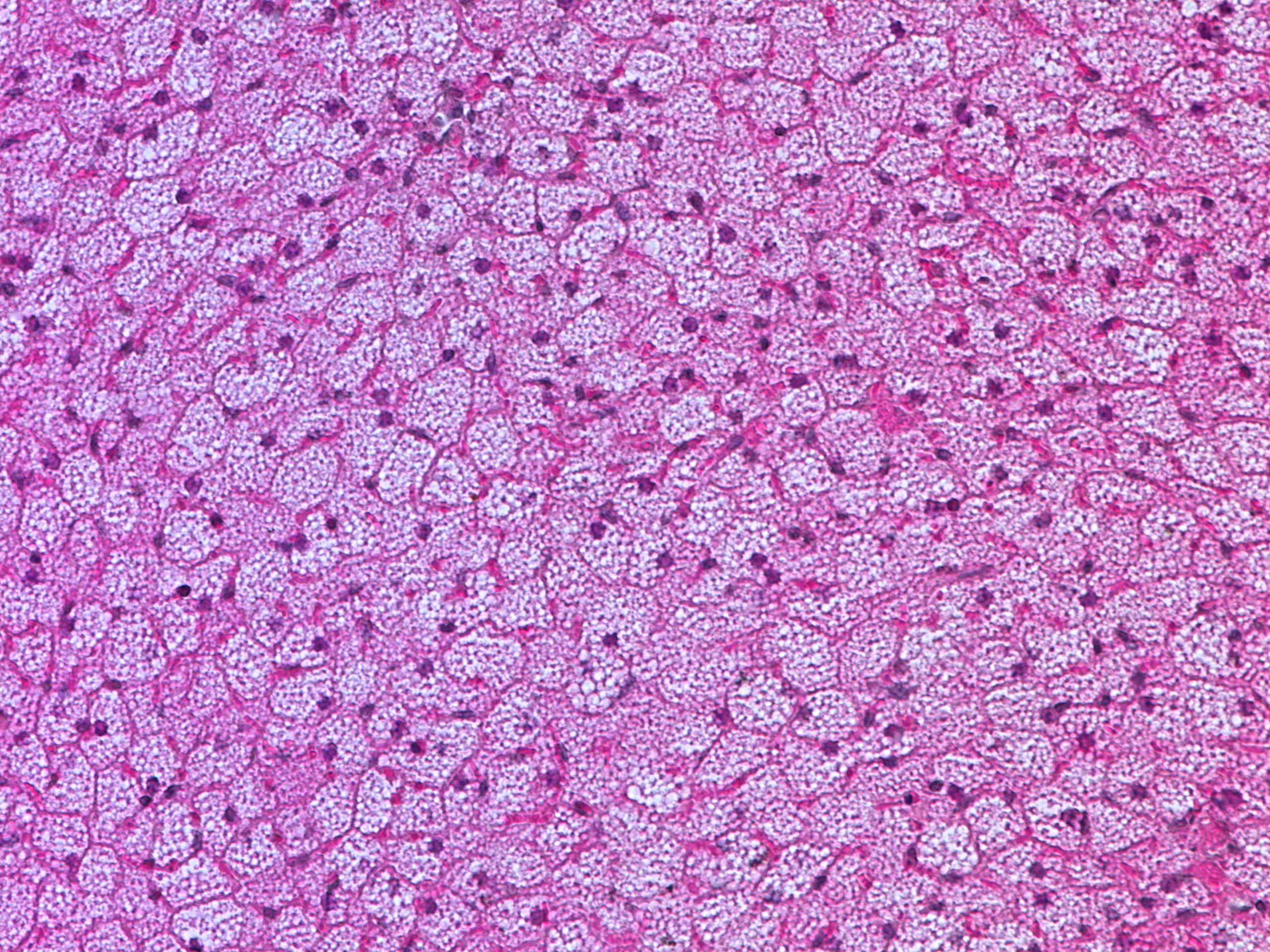

Supplement: Supplementary file 10 — Source data Fig. 5 [file 44319_2025_642_MOESM10_ESM.zip › Figure 5/5F/P2xr4flfl-LysM-Cre+ HE.tif]

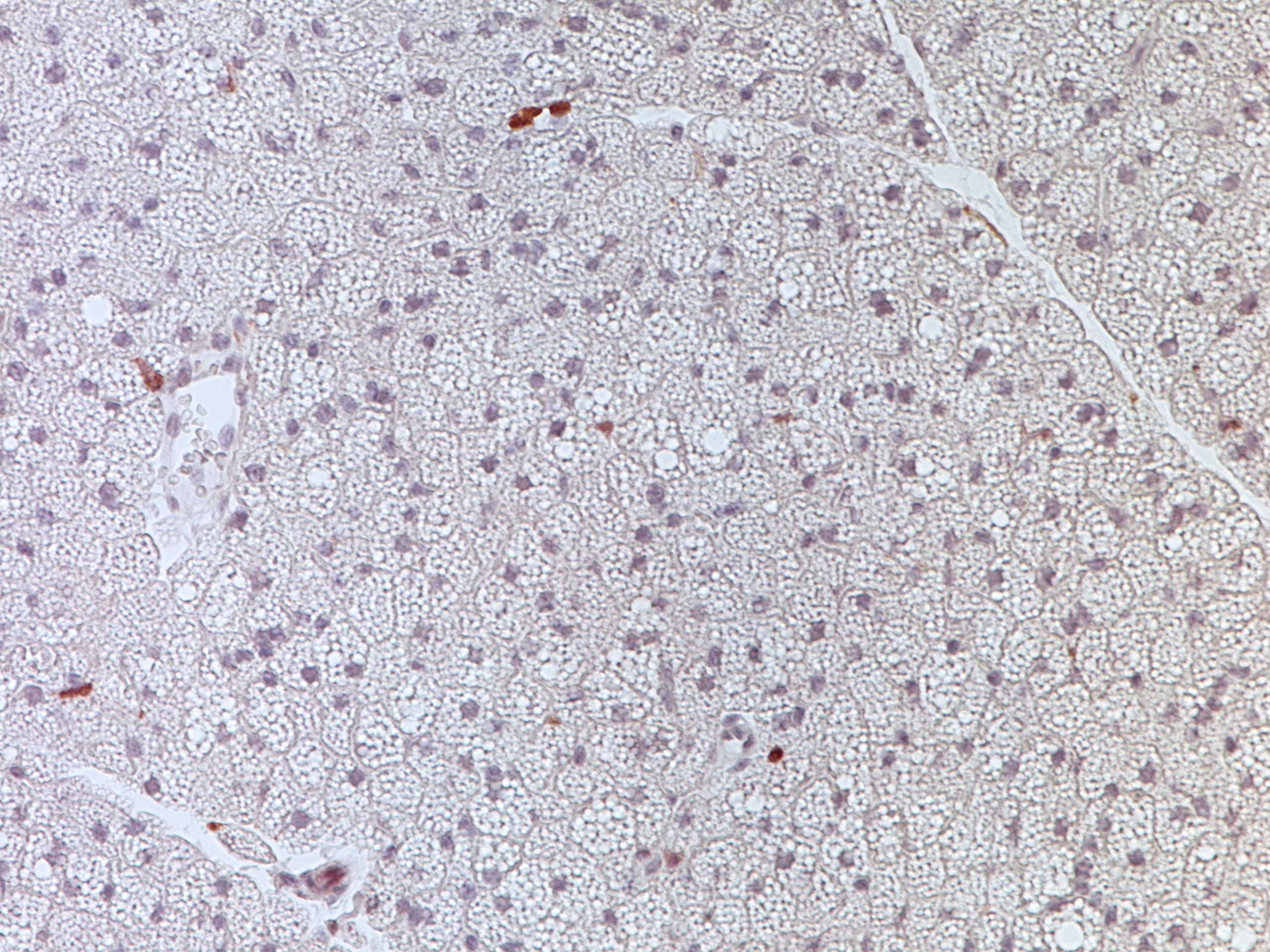

Supplement: Supplementary file 10 — Source data Fig. 5 [file 44319_2025_642_MOESM10_ESM.zip › Figure 5/5F/P2xr4flfl-LysM-Cre+ MAC2.tif]

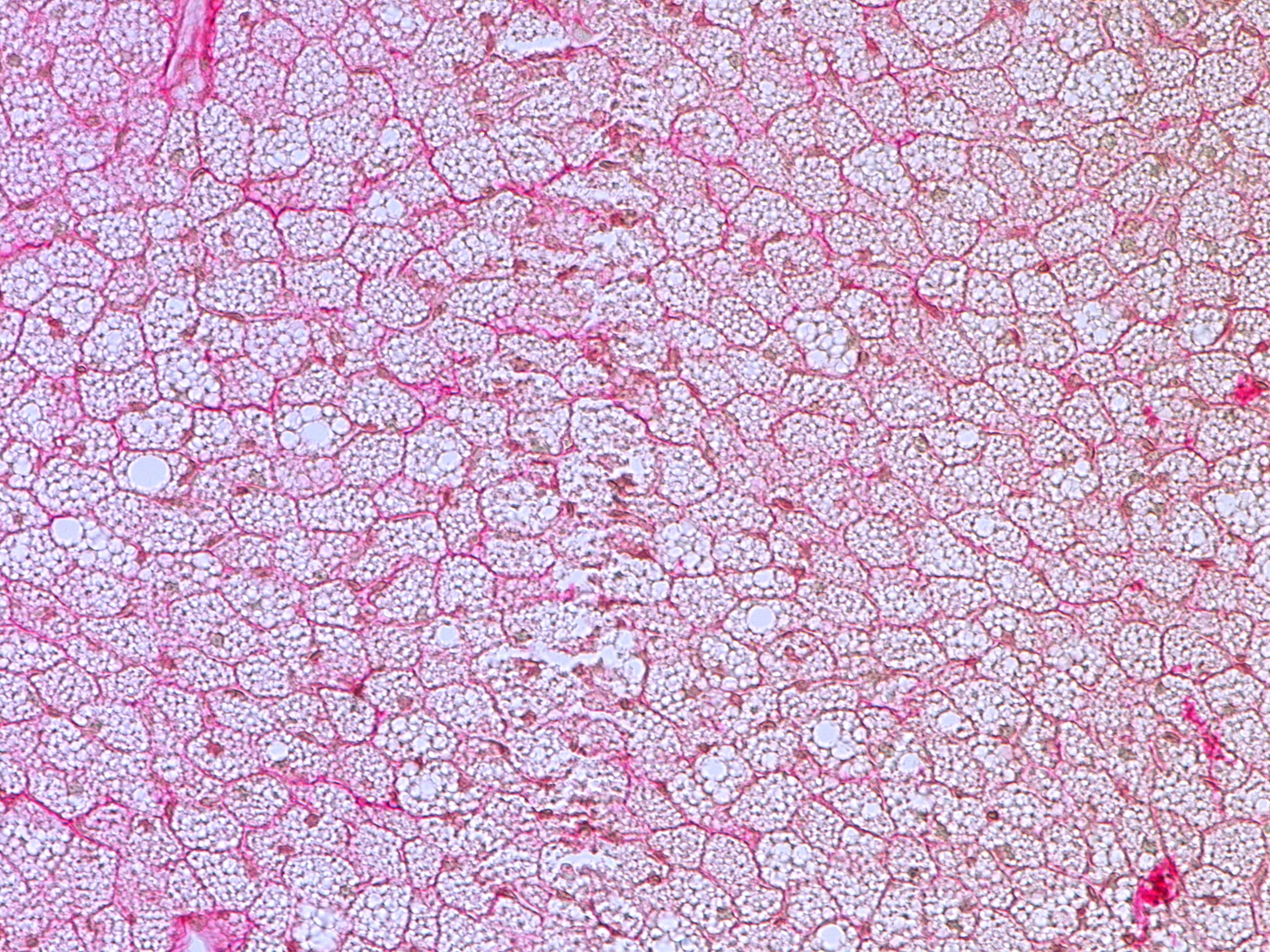

Supplement: Supplementary file 10 — Source data Fig. 5 [file 44319_2025_642_MOESM10_ESM.zip › Figure 5/5F/P2xr4flfl-LysM-Cre+ Sirius Red.tif]

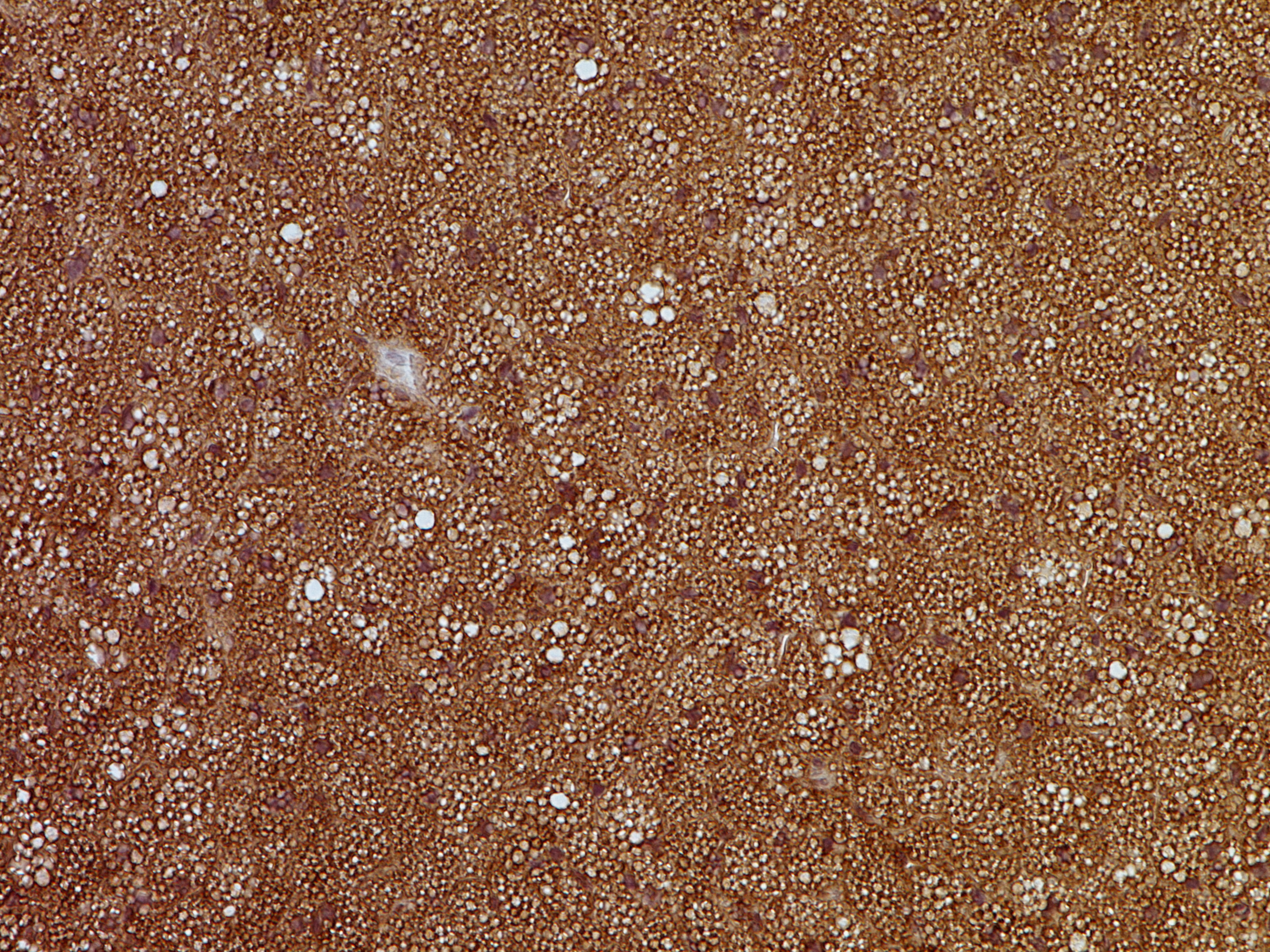

Supplement: Supplementary file 10 — Source data Fig. 5 [file 44319_2025_642_MOESM10_ESM.zip › Figure 5/5F/P2xr4flfl-LysM-Cre+ UCP1.tif]

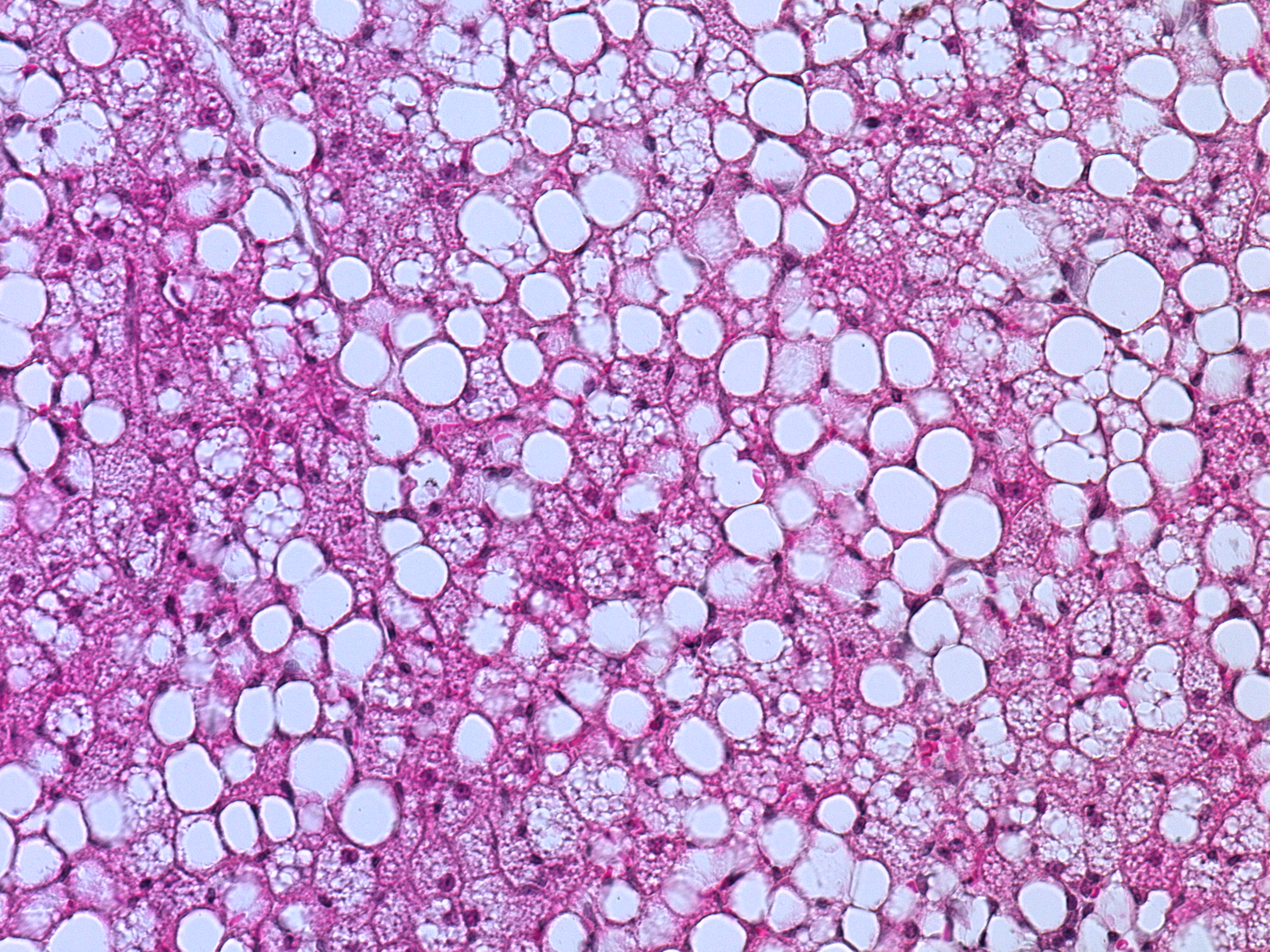

Supplement: Supplementary file 10 — Source data Fig. 5 [file 44319_2025_642_MOESM10_ESM.zip › Figure 5/5F/P2xr4flfl-LysM-Cre- HE.tif]

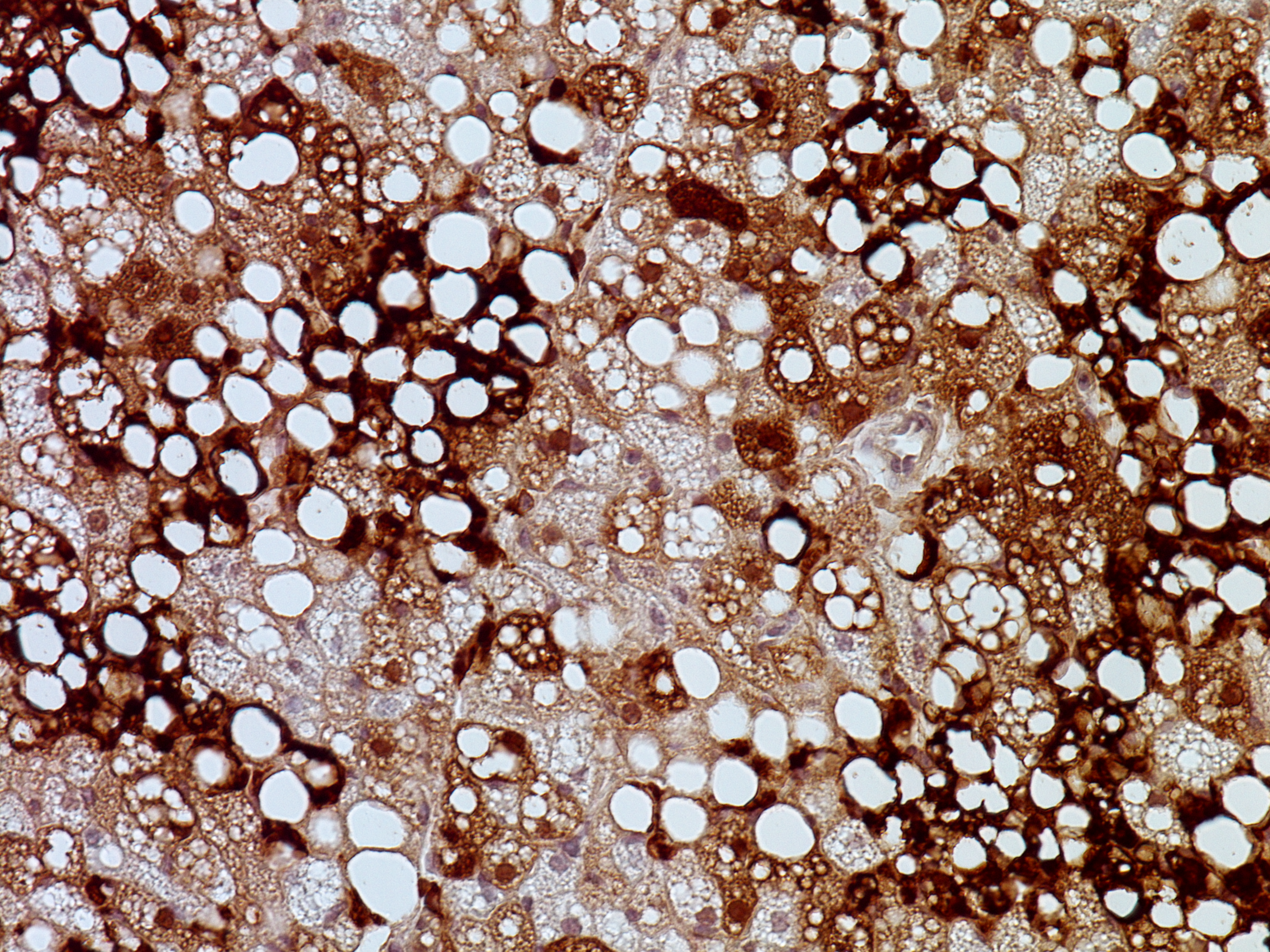

Supplement: Supplementary file 10 — Source data Fig. 5 [file 44319_2025_642_MOESM10_ESM.zip › Figure 5/5F/P2xr4flfl-LysM-Cre- MAC2.tif]

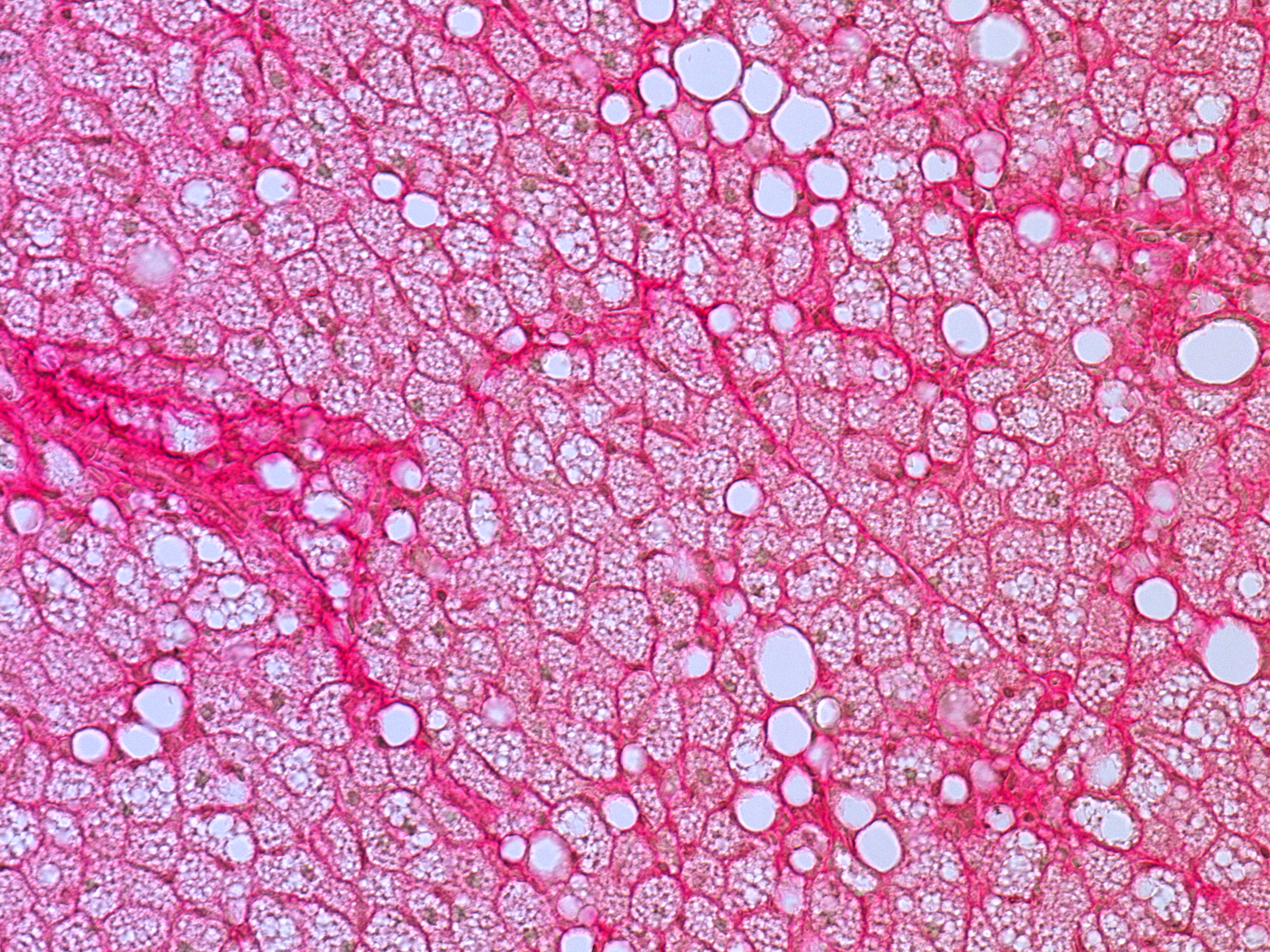

Supplement: Supplementary file 10 — Source data Fig. 5 [file 44319_2025_642_MOESM10_ESM.zip › Figure 5/5F/P2xr4flfl-LysM-Cre- Sirius Red.tif]

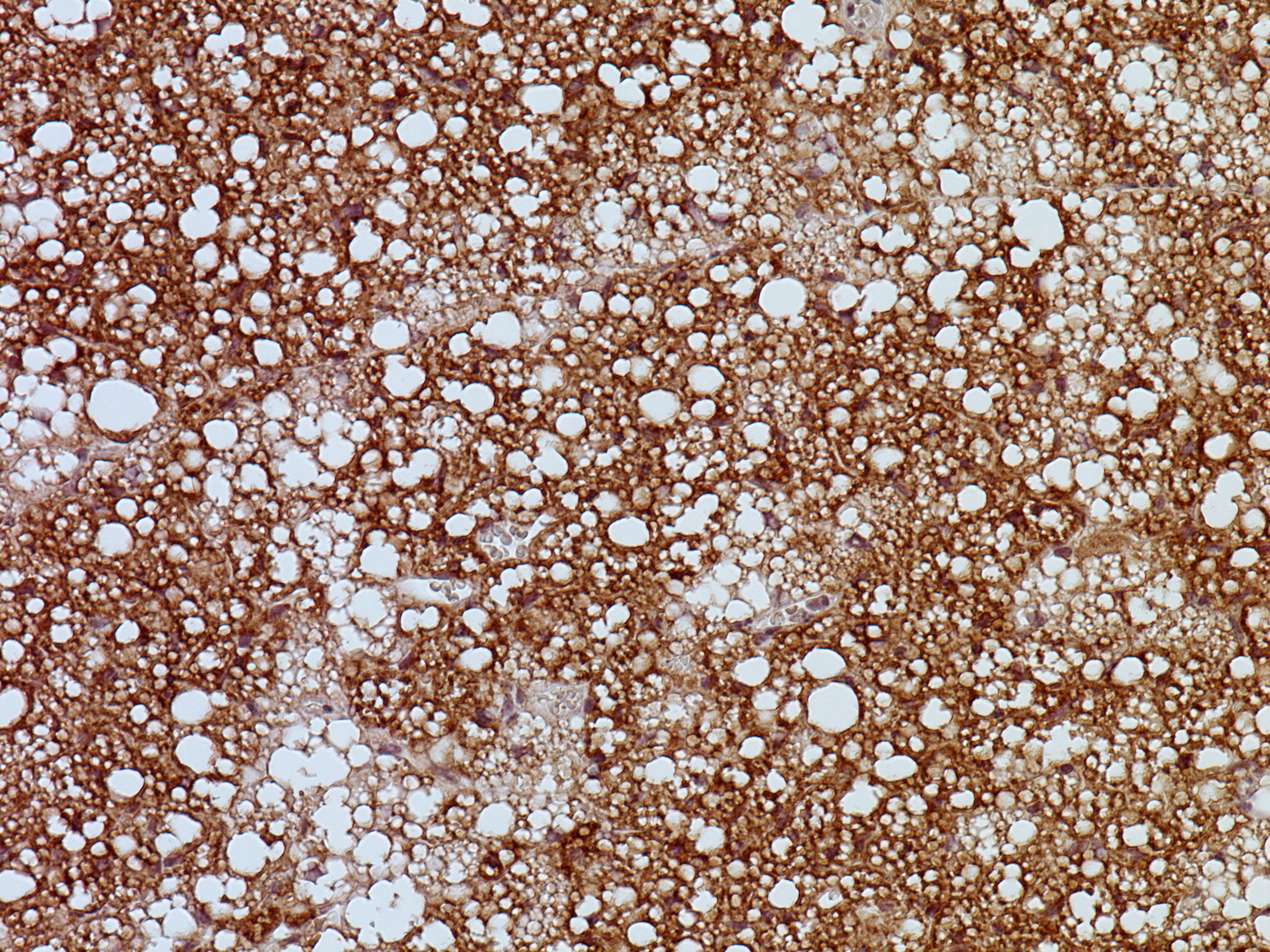

Supplement: Supplementary file 10 — Source data Fig. 5 [file 44319_2025_642_MOESM10_ESM.zip › Figure 5/5F/P2xr4flfl-LysM-Cre- UCP1.tif]

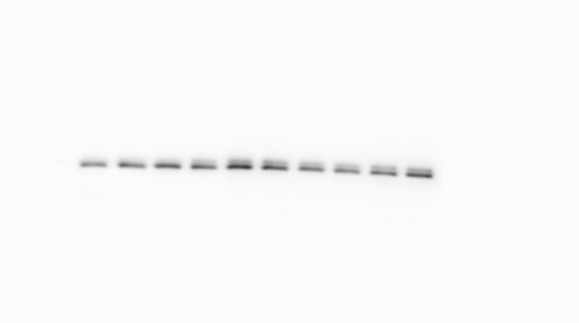

Supplement: Supplementary file 10 — Source data Fig. 5 [file 44319_2025_642_MOESM10_ESM.zip › Figure 5/5G/g-Tubulin.jpg]

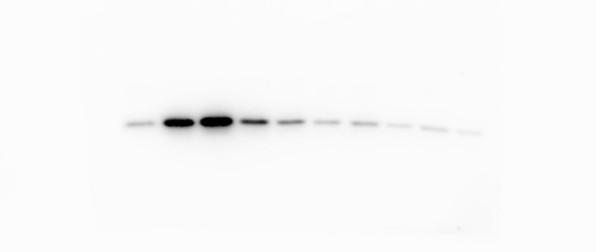

Supplement: Supplementary file 10 — Source data Fig. 5 [file 44319_2025_642_MOESM10_ESM.zip › Figure 5/5G/MAC2.jpg]

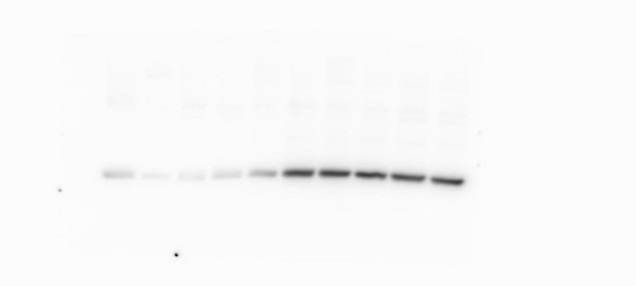

Supplement: Supplementary file 10 — Source data Fig. 5 [file 44319_2025_642_MOESM10_ESM.zip › Figure 5/5G/UCP1.jpg]

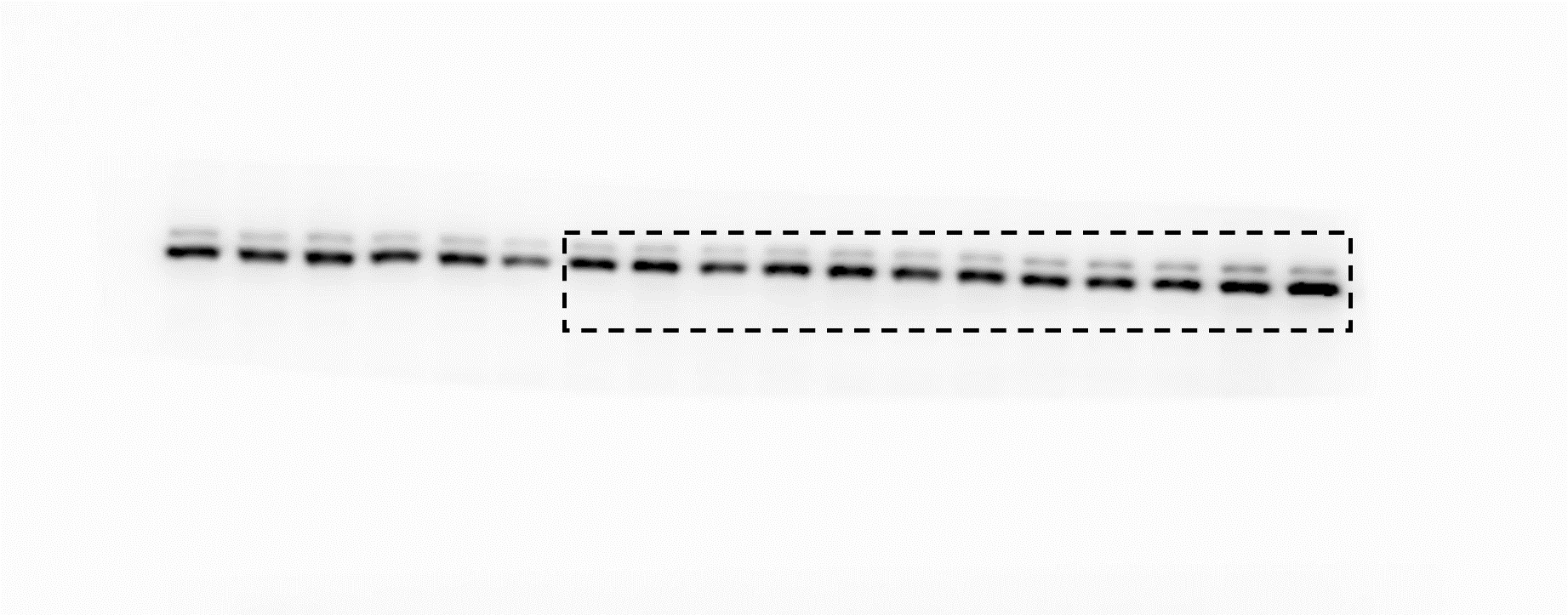

Supplement: Supplementary file 11 — Source data Fig. 6 [file 44319_2025_642_MOESM11_ESM.zip › Figure 6/6F/g-Tubulin.png]

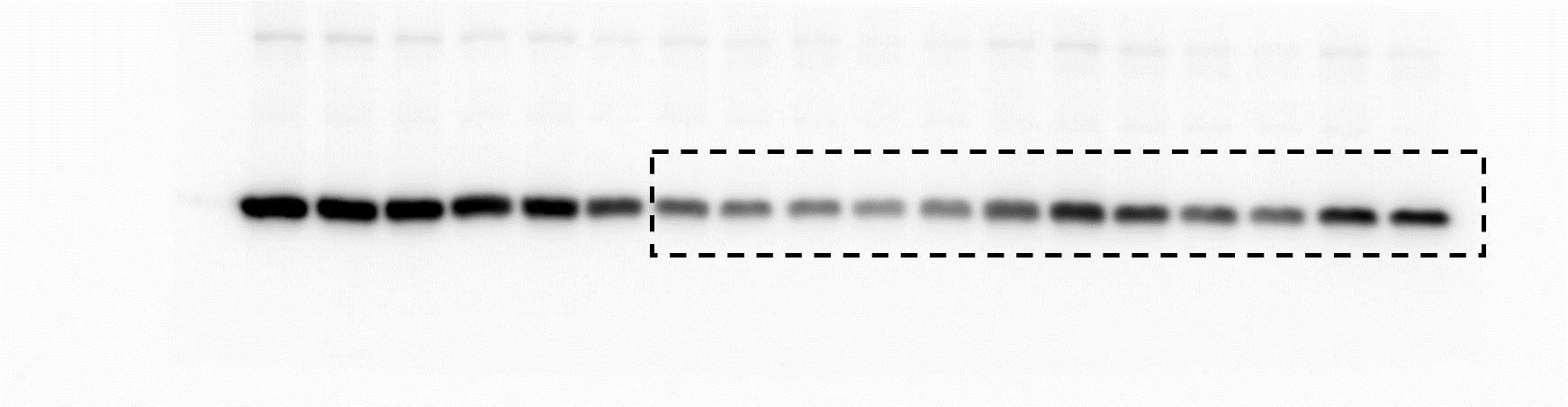

Supplement: Supplementary file 11 — Source data Fig. 6 [file 44319_2025_642_MOESM11_ESM.zip › Figure 6/6F/UCP1.png]

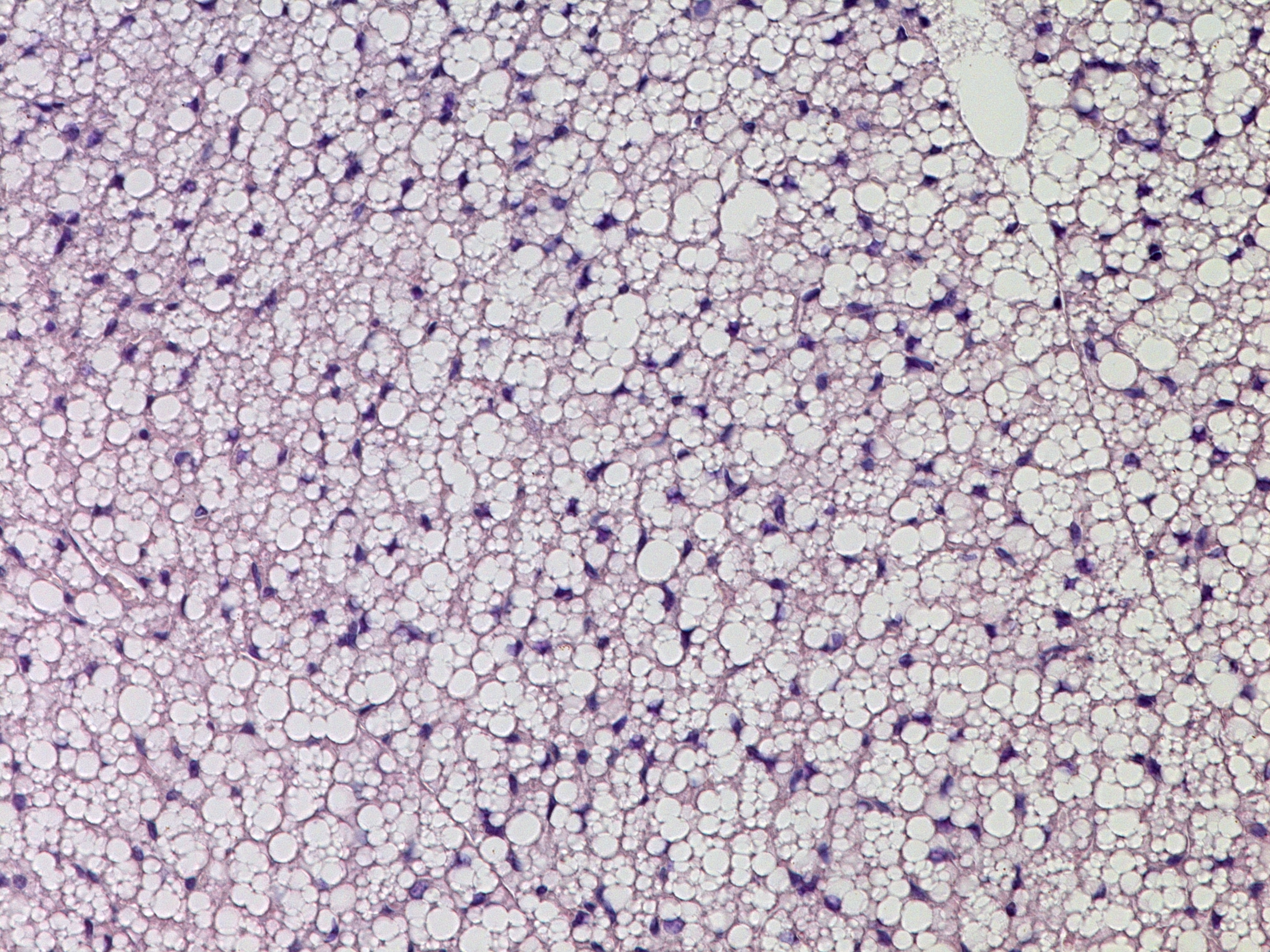

Supplement: Supplementary file 11 — Source data Fig. 6 [file 44319_2025_642_MOESM11_ESM.zip › Figure 6/6H/control Thermoneutrality HE.tif]

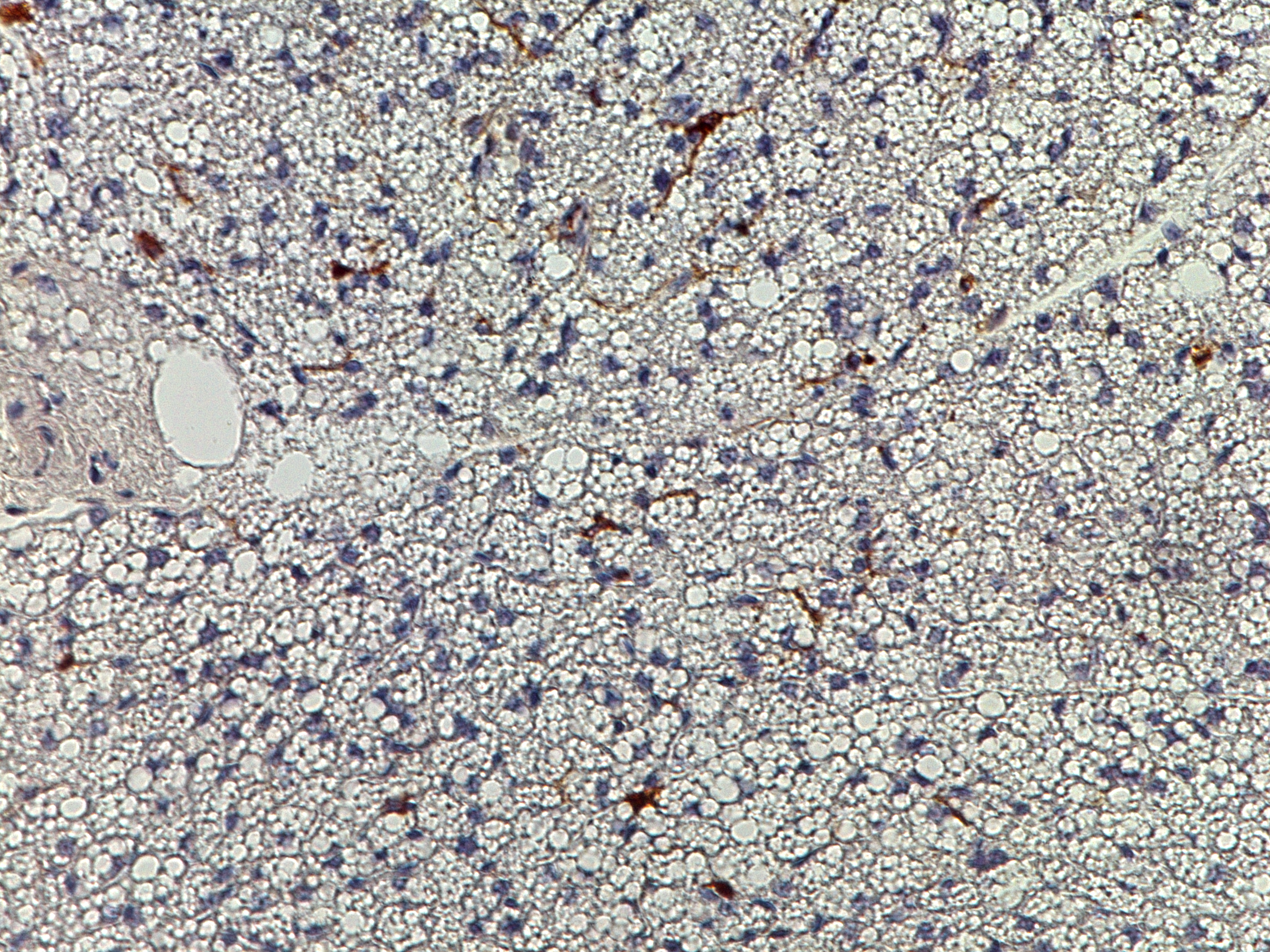

Supplement: Supplementary file 11 — Source data Fig. 6 [file 44319_2025_642_MOESM11_ESM.zip › Figure 6/6H/control Thermoneutrality MAC2.tif]

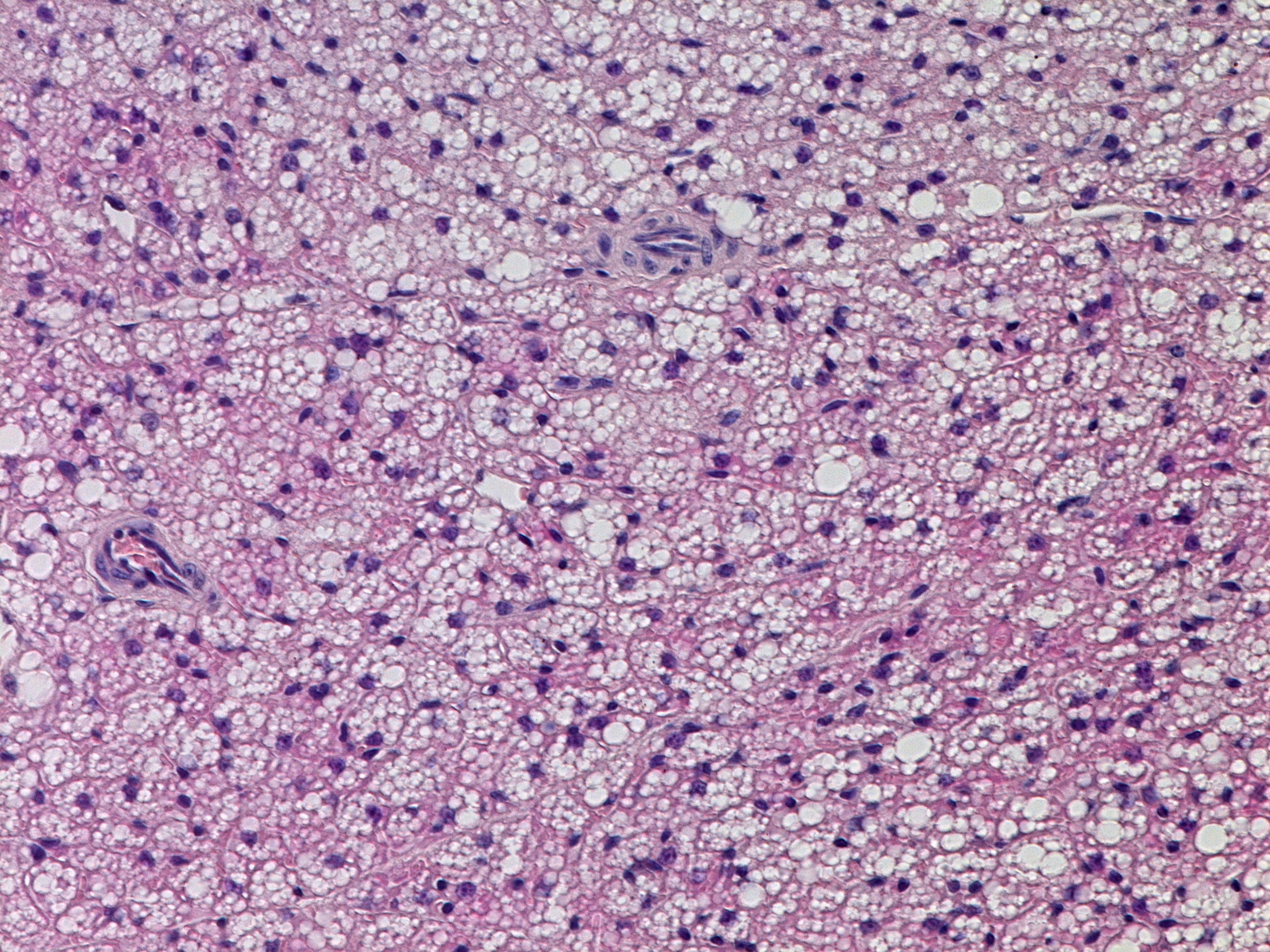

Supplement: Supplementary file 11 — Source data Fig. 6 [file 44319_2025_642_MOESM11_ESM.zip › Figure 6/6H/P2X4KO + P2X7NB HE.tif]

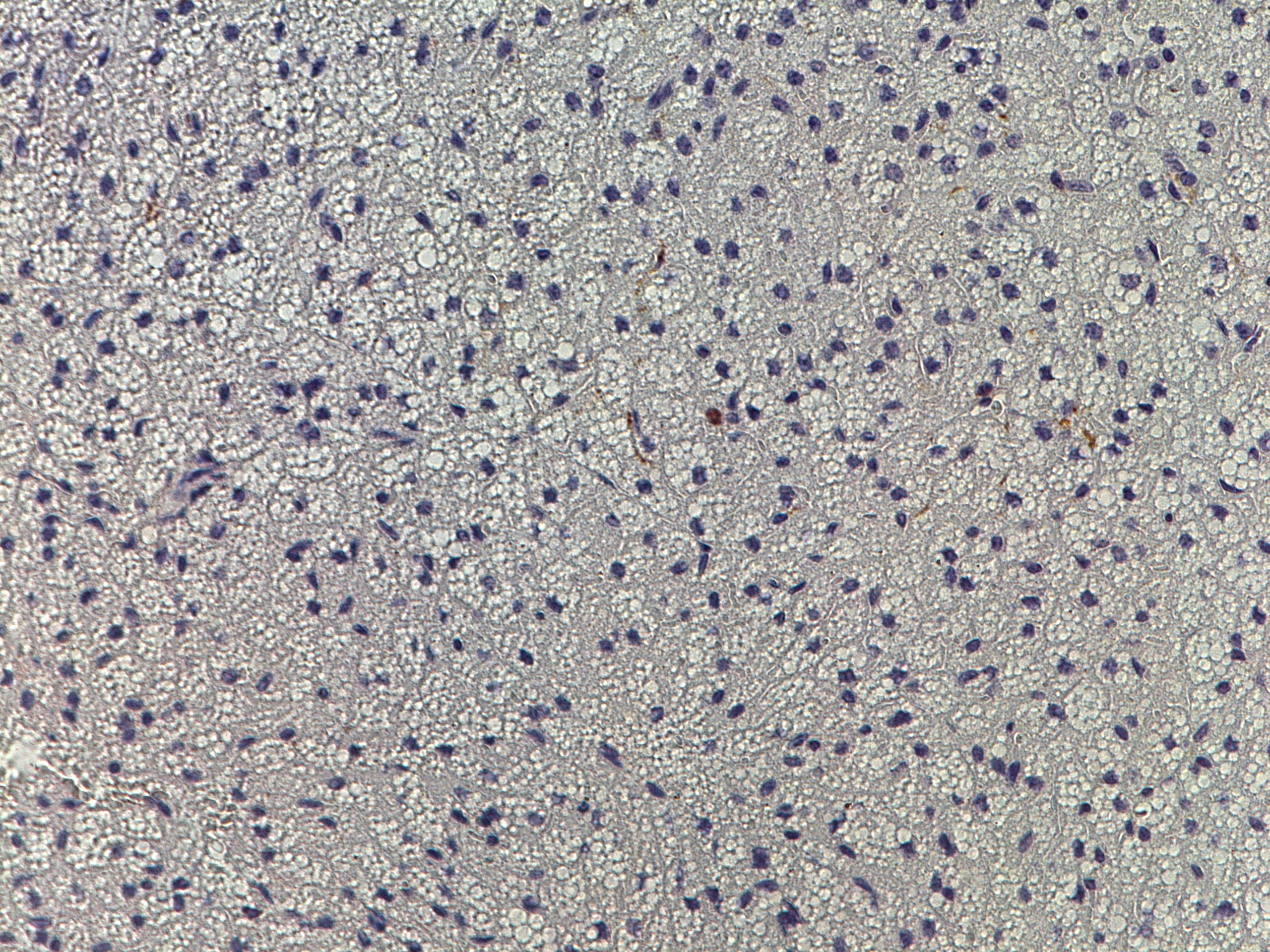

Supplement: Supplementary file 11 — Source data Fig. 6 [file 44319_2025_642_MOESM11_ESM.zip › Figure 6/6H/P2X4KO + P2X7NB Thermoneutrality MAC2.tif]

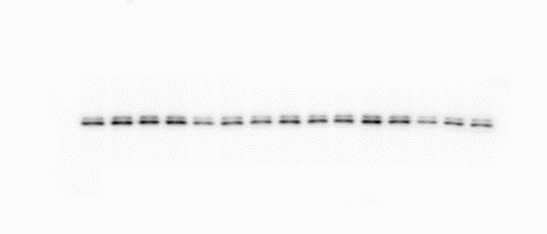

Supplement: Supplementary file 11 — Source data Fig. 6 [file 44319_2025_642_MOESM11_ESM.zip › Figure 6/6Q/g-Tubulin.png]

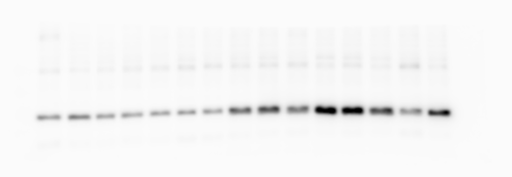

Supplement: Supplementary file 11 — Source data Fig. 6 [file 44319_2025_642_MOESM11_ESM.zip › Figure 6/6Q/UCP1.tif]
