# Supplementary material for: Purinergic adipocyte-macrophage crosstalk promotes degeneration of thermogenic brown adipose tissue
Source: EMBO Rep. 2025 Nov 19;26(24):6460–93. doi: 10.1038/s44319-025-00642-y (PMC12715258; doi:10.1038/s44319-025-00642-y)
Supplement: Supplementary file 12 — Expanded View Figures [file 44319_2025_642_MOESM12_ESM.pdf]

Expanded View Figures

Figure EV1. Related to Fig. 1. Inflammatory BAT remodeling is triggered by imbalanced BAT activation.

(**A–G**) Male *Ucp1*$^{-/-}$ mice and wild-type (WT) littermates were housed at 22 °C. (**A**) iBAT weight ($n = 5$). (*P = 0.0004). (**B**) BAT gene expression of *Ucp1*, fibrosis and inflammatory marker genes ($n = 5$). (*Ucp1*: *P = 2.0040E-05; *Tnf*: *P = 0.0054; *Ccl2*: *P = 0.0275; *Il1b*: *P = 0.0703; *Ifng*: *P = 0.0117; *Emr1*: *P = 0.0044; *Cd4*: *P = 0.0064; *Cd8b1*: *P = 0.0055; *Timp1*: *P = 0.0172; *Col1a1*: *P = 0.0091; left to right). (**C**) Body weight ($n = 5$). (**D**) BAT images of HE and Sirius Red stainings as well as immunostaining of MAC2. Scale bar, 50 µm. ($n = 4$). For better comparison, images of all individual mice are presented here, which includes the representative image shown in the main Fig. 1. (**E**) Quantification of lipid droplet area as shown in (**D**) ($n = 4$). (**F**) Quantification of MAC2 staining as shown in (**D**) ($n = 4$). (*P = 0.0038). (**G**) Quantification of Sirius red staining as shown in (**D**) ($n = 4$). (*P = 0.0077). (**H**) Male *Ucp1*$^{-/-}$ mice and wild-type (WT) littermates were housed at 22 °C or 6 °C. Western Blot of BAT ($n = 5$). (**I**) Quantification of Western blot shown in (**H**). (UCP1: *P = 0.0032; #P = 0.0016; *P = <0.0001; MAC2: *P = 0.0136; *P = 0.0003; left to right). (**J**) Quantification of Western blot shown in Fig. 1C. (UCP1: *P = <0.0001; #P = <0.0001; *P = <0.0001; MAC2: *P = 0.0001; #P = 0.0005; TH: *P = 0.0037; #P = <0.0001; *P = <0.0001; left to right). Data are presented as mean values ± SEM. *$P < 0.05$ by Student's *t* test comparing WT vs. *Ucp1*$^{-/-}$-mice (**A–C**, **E–G**) or two-way ANOVA comparing WT vs. *Ucp1*$^{-/-}$-mice (**I**, **J**). #$P < 0.05$ by two-way ANOVA comparing 22 °C vs 6 °C (**I**) or sham vs DNV (**J**). *N* values indicate biological replicates.

© The Author(s)

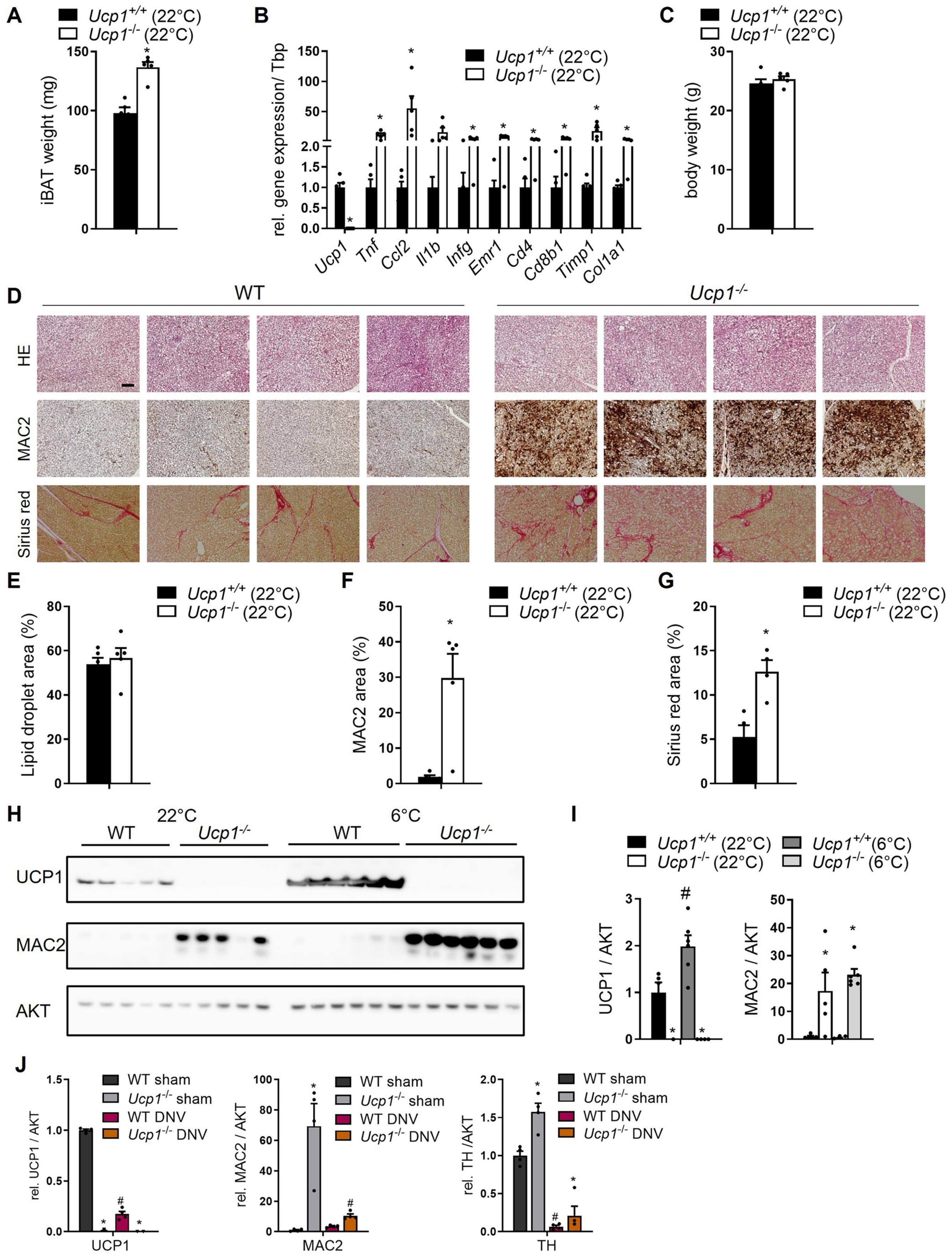
A
Ucp1+/+ (22°C)
Ucp1-/- (22°C)
iBAT weight (mg)
B
rel. gene expression/ Tbp
Ucp1 Tnf Ccl2 Il1b Infg Emr1 Cd4 Cd8b1 Timp1 Col1a1
C
body weight (g)
D
WT
Ucp1-/-
HE
MAC2
Sirius red
E
Lipid droplet area (%)
F
MAC2 area (%)
G
Sirius red area (%)
H
22°C
6°C
WT
Ucp1-/-
UCP1
MAC2
AKT
I
Ucp1+/+(6°C)
Ucp1-/- (6°C)
UCP1 / AKT
MAC2 / AKT
J
WT sham
Ucp1-/- sham
WT DNV
Ucp1-/- DNV
rel. UCP1 / AKT
rel. MAC2 / AKT
rel. TH /AKT
UCP1
MAC2
TH

 © The Author(s)

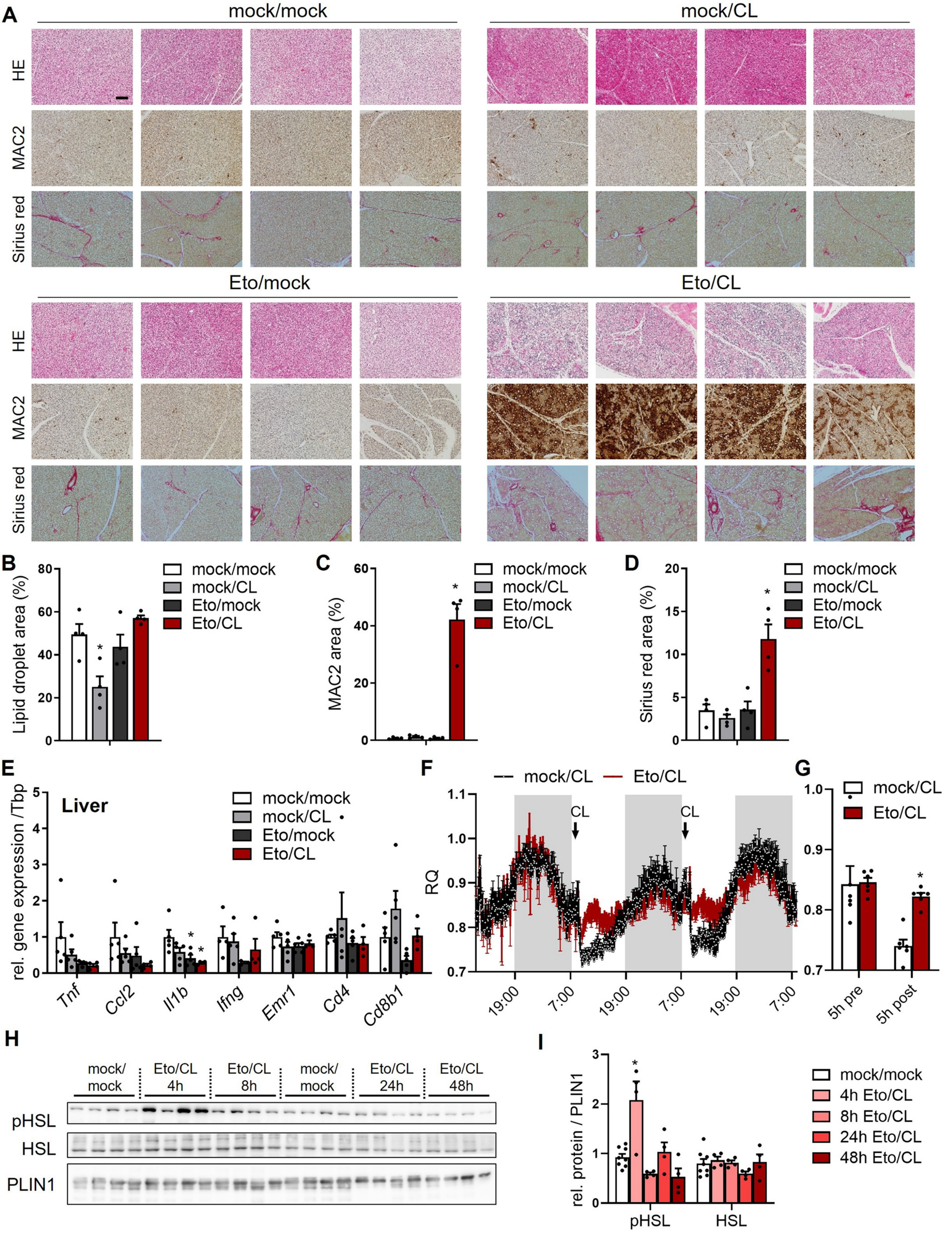
A
mock/mock
mock/CL
Eto/mock
Eto/CL
HE
MAC2
Sirius red
B
Lipid droplet area (%)
mock/mock
mock/CL
Eto/mock
Eto/CL
C
MAC2 area (%)
D
Sirius red area (%)
E
rel. gene expression /Tbp
Liver
Tnf
Ccl2
Il1b
Ifng
Emr1
Cd4
Cd8b1
F
mock/CL
Eto/CL
RQ
CL
19:00
7:00
G
5h pre
5h post
H
mock/ mock
Eto/CL 4h
Eto/CL 8h
Eto/CL 24h
Eto/CL 48h
pHSL
HSL
PLIN1
I
rel. protein / PLIN1
4h Eto/CL
8h Eto/CL
24h Eto/CL
48h Eto/CL

Figure EV2. Related to Fig. 1. Inflammatory BAT remodeling is triggered by imbalanced BAT activation.

Wild-type mice housed at 22 °C were injected daily for three subsequent days with either vehicle (mock/mock), with the beta-3-adrenergic agonist CL316,243 alone (mock/CL), with the inhibitor of beta oxidation etomoxir alone (Eto/mock) or with the combination of both (Eto/CL). (**A**) Representative images of BAT of individual mice employing HE, Sirius Red and MAC2 (immune)-stainings, respectively. Scale bar, 50 µm. ($n = 4$). For better comparison, images of all individual mice are presented here, which includes the representative image shown in the main Fig. 1. (**B**) Quantification of lipid droplet area ($n = 4$, 3 images per section per mouse). (*$P = 0.0073$). (**C**) Quantification of MAC2 staining as shown in (**A**) ($n = 4$, 3 images per section per mouse). (*$P = <0.0001$). (**D**) Quantification of Sirius red staining as shown in (**A**) ($n = 4$, 3 images per section per mouse). (*$P = 0.0004$). (**E**) Liver gene expression of inflammatory marker genes ($n = 4$–5). (*$P = 0.0246$; *$P = 0.0095$; left to right). (**F**) Respiratory quotient (RQ) in mock/CL and Eto/CL mice. The first and second CL injection are indicated by arrows ($n = 6$). (**G**) Quantification of respiratory quotient for 5 h pre and post CL injection ($n = 6$). (*$P = 4.6982E-05$). (**H**) Wild-type mice were treated with Eto and CL for indicated time spans. Western Blot of Plin1, HSL and phospho HSL (pHSL) in WAT samples ($n = 4$). (**I**) Quantification of Western blot shown in (**H**). (*$P = 0.0004$). Data are presented as mean values ± SEM. *$P < 0.05$ by ANOVA (**B**–**E**, **I**) compared to mock control or Student's *t* test (**G**). *N* values indicate biological replicates.

 © The Author(s)

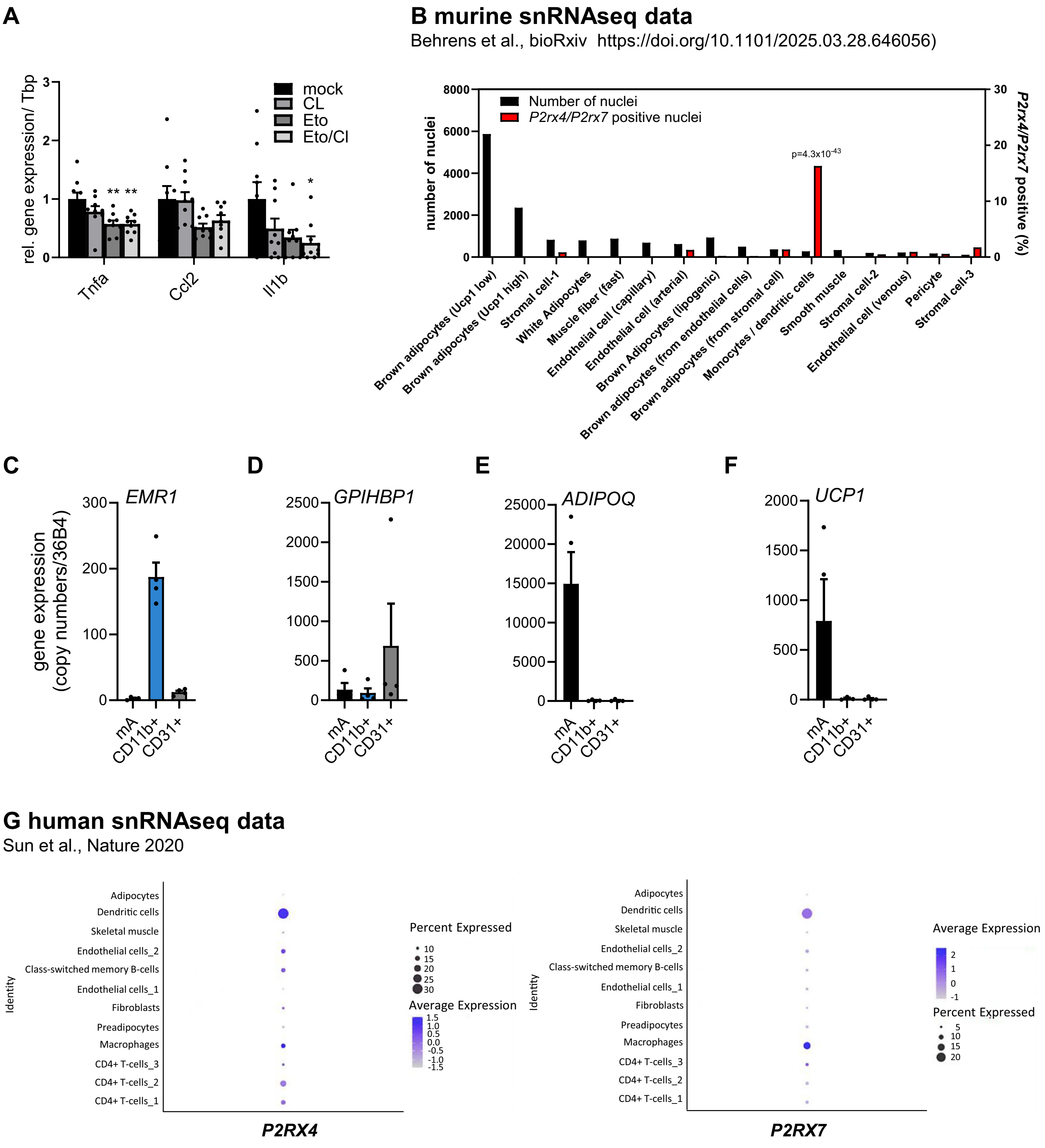
A
rel. gene expression/ Tbp
mock
CL
Eto
Eto/Cl
Tnfa
Ccl2
Il1b
B murine snRNAseq data
Behrens et al., bioRxiv https://doi.org/10.1101/2025.03.28.646056)
Number of nuclei
P2rx4/P2rx7 positive nuclei
number of nuclei
P2rx4/P2rx7 positive (%)
p=4.3x10-43
Brown adipocytes (Ucp1 low)
Brown adipocytes (Ucp1 high)
Stromal cell-1
White Adipocytes
Muscle fiber (fast)
Endothelial cell (capillary)
Endothelial cell (arterial)
Brown Adipocytes (lipogenic)
Brown adipocytes (from endothelial cells)
Brown adipocytes (from stromal cell)
Monocytes / dendritic cells
Smooth muscle
Stromal cell-2
Endothelial cell (venous)
Pericyte
Stromal cell-3
C
D
E
F
EMR1
GPIHBP1
ADIPOQ
UCP1
gene expression (copy numbers/36B4)
mA
CD11b+
CD31+
G human snRNAseq data
Sun et al., Nature 2020
Identity
Adipocytes
Dendritic cells
Skeletal muscle
Endothelial cells_2
Class-switched memory B-cells
Endothelial cells_1
Fibroblasts
Preadipocytes
Macrophages
CD4+ T-cells_3
CD4+ T-cells_2
CD4+ T-cells_1
Percent Expressed
Average Expression
P2RX4
P2RX7

◀ **Figure EV3. Related to Fig. 3. Brown adipocyte dysfunction causes ATP secretion and is linked to purinergic receptor signaling in BAT.**

(**A**) Brown adipocytes differentiated from BAT stromal vascular fractions were treated for 24 h with or without Eto (25 µM) in the presence or absence of CL (50 nM). Gene expression of cytokines ($n = 9$). (*$P = 0.0053$; *$P = 0.0054$; *$P = 0.0393$, left to right). (**B**) Analysis of published murine snRNAseq data (Behrens et al, Mol Metab. 2025 Nov;101:102252. doi: 10.1016/j.molmet.2025.102252.). Presentation of *P2rx4/P2rx7*-double positive nuclei in a dataset of murine single nucleus RNA sequencing. Cell types with a nuclei count above 100 were re-analyzed. (**C–F**) Human BAT samples from four donors were sorted using low speed centrifugation to enrich mature adipocytes (mA), followed by MACS® to isolate CD31+ endothelial cells and CD11b+ myeloid cells. (**C**) Gene expression of the macrophage marker *EMR1* in the three fractions ($n = 4$). (**D**) Gene expression of the endothelial cell marker *GPIHBP1* in the three fractions ($n = 4$). (**E**) Gene expression of the adipocyte marker *ADIPOQ* in the three fractions ($n = 4$). (**F**) Gene expression of the thermogenic brown adipocyte marker *UCP1* in the three fractions ($n = 4$). (**G**) Analysis of published human snRNAseq data (Sun et al, Nature 2020). Expression of *P2RX4/P2RX7* in various cell clusters of human brown adipose tissue. Data are presented as mean values ± SEM. *$P < 0.05$ by ANOVA compared to mock control (**A**, **C–F**). *N* values indicate biological replicates.

© The Author(s)

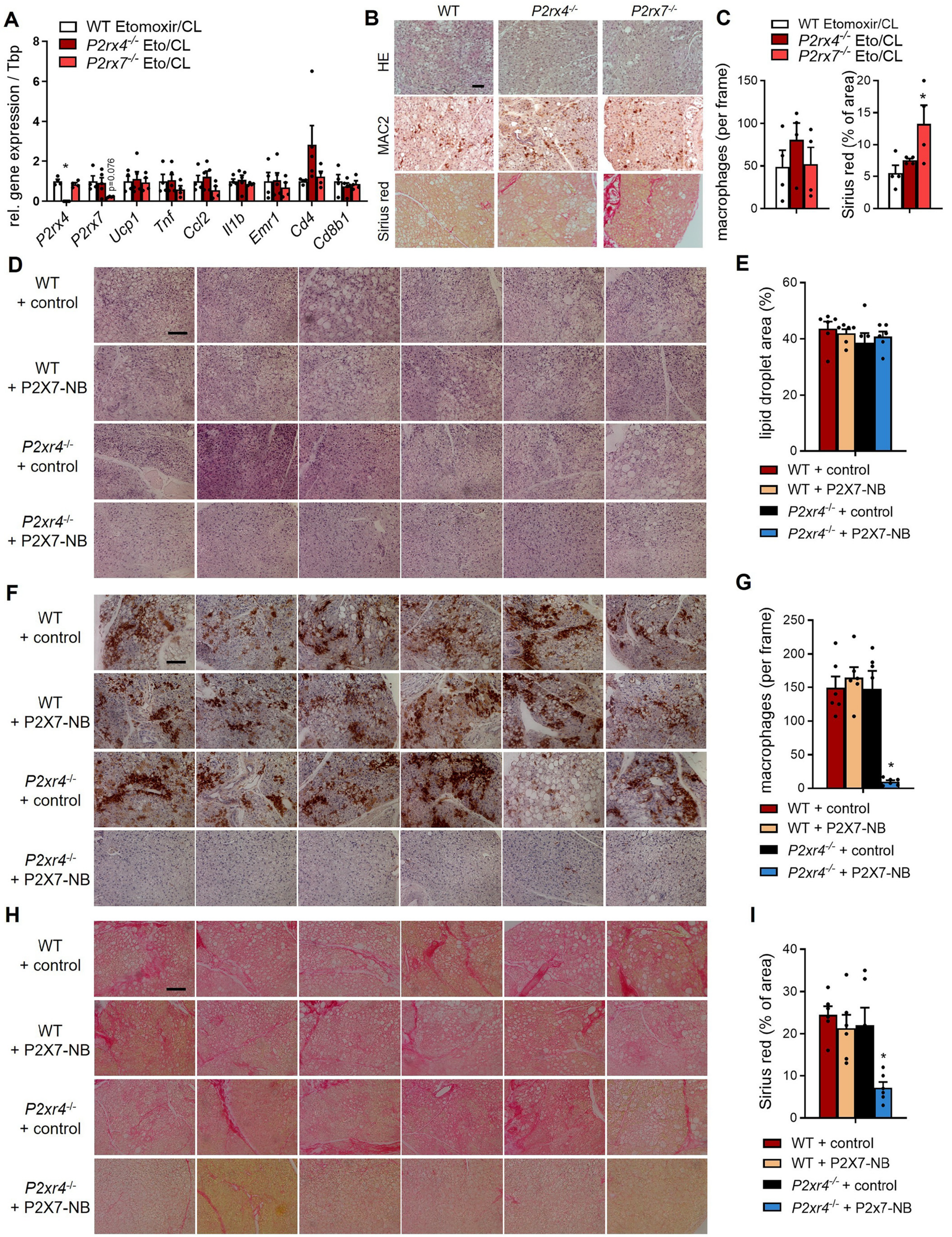

A
WT Etomoxir/CL
P2rx4-/- Eto/CL
P2rx7-/- Eto/CL
rel. gene expression / Tbp
p=0.076
P2rx4
P2rx7
Ucp1
Tnf
Ccl2
Il1b
Emr1
Cd4
Cd8b1
B
WT
P2rx4-/-
P2rx7-/-
HE
MAC2
Sirius red
C
macrophages (per frame)
Sirius red (% of area)
D
WT + control
WT + P2X7-NB
P2rx4-/- + control
P2rx4-/- + P2X7-NB
E
lipid droplet area (%)
F
G
macrophages (per frame)
H
I
Sirius red (% of area)
P2xr4-/- + P2x7-NB

Figure EV4. Related to Fig. 4. Combined inhibition of P2X4 and P2X7 prevents pharmacologically induced BAT degeneration thereby preserving thermogenic capacity.

(**A–C**) Wild-type (WT), *P2rx4*$^{-/-}$ and *P2rx7*$^{-/-}$ mice were daily injected with Eto and CL on two consecutive days. (**A**) BAT gene expression of *Ucp1*, purinergic receptors and inflammatory markers ($n = 4$–5). (*P = <0.0001). (**B**) Representative BAT images of HE, Sirius Red and MAC2 (immune)-stainings. Scale bar, 50 µm. (**C**) Quantification of macrophages and Sirius red staining ($n = 4$, 3 images per section per mouse). (*$P = 0.0284$). (**D–I**) Wild-type (WT) and *P2rx4*$^{-/-}$ mice were pretreated with the P2X7-inhibiting nanobody or vehicle (control). Then, mice were daily injected with Eto and CL on two consecutive days. (**D**) BAT images of HE staining of individual mice. Scale bar, 50 µm. For better comparison, images of all individual mice are presented here, which includes the representative image shown in the main Fig. 4. (**E**) Quantification of lipid droplet area ($n = 6$, 3 images per section per mouse). (**F**) BAT images of MAC2 immunestaining of individual mice. Scale bar, 50 µm. For better comparison, images of all individual mice are presented here, which includes the representative image shown in the main Fig. 4. (**G**) Quantification of macrophages ($n = 6$, 3 images per section per mouse). (*P = <0.0001). (**H**) BAT images of Sirius Red staining of individual mouse. Scale bar, 50 µm. For better comparison, images of all individual mice are presented here, which includes the representative image shown in the main Fig. 4. (**I**) Quantification of Sirius Red staining ($n = 6$, 3 images per section per mouse). (*$P = 0.0012$). Data are presented as mean values ± SEM. *$P < 0.05$ by ANOVA compared to WT Eto/CL (**A**, **C**) or WT + control (**E**, **G**, **I**). *N* values indicate biological replicates.

 © The Author(s)

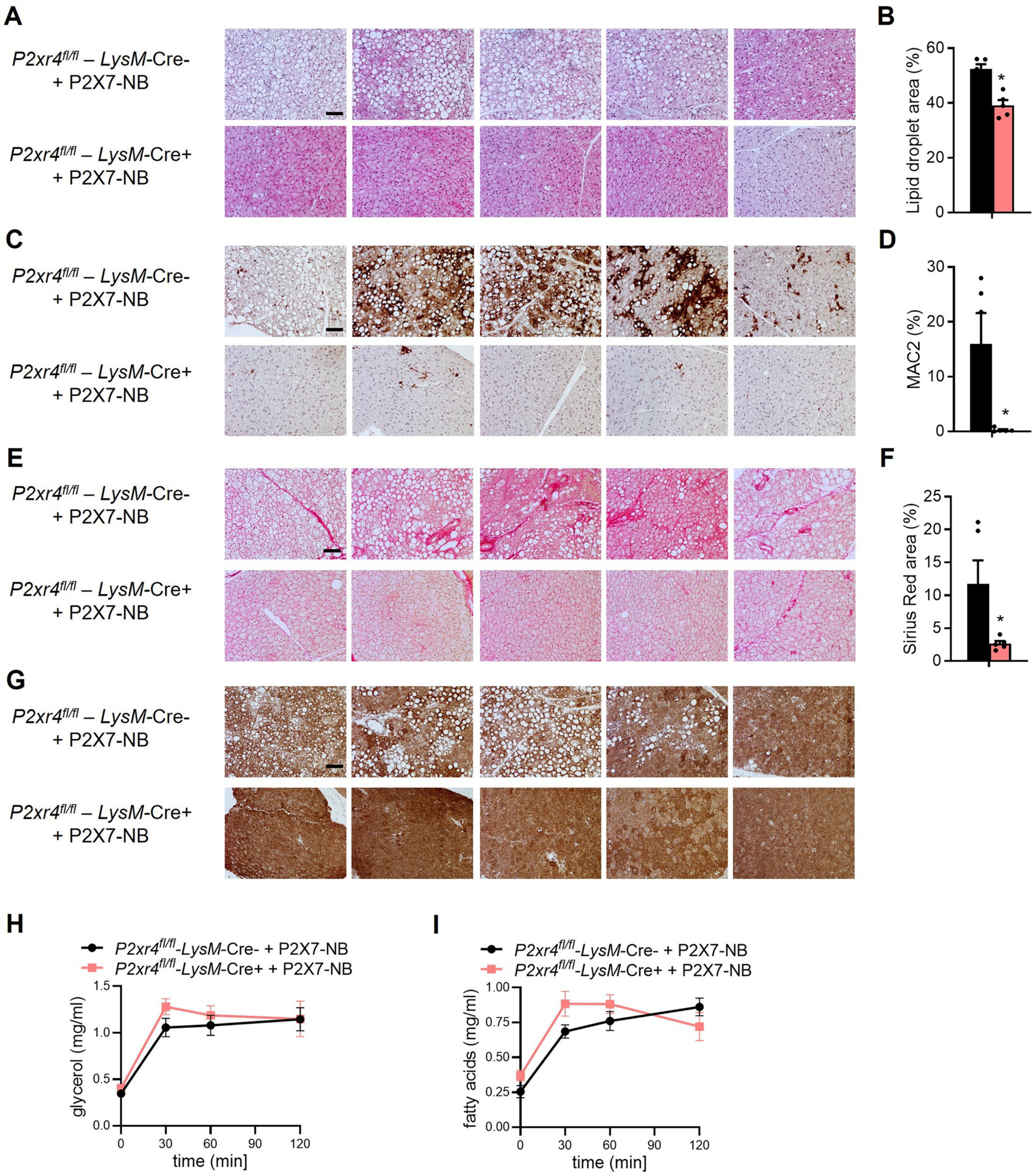
A
P2xr4fl/fl – LysM-Cre-
+ P2X7-NB
P2xr4fl/fl – LysM-Cre+
+ P2X7-NB
B
Lipid droplet area (%)
C
D
MAC2 (%)
E
F
Sirius Red area (%)
G
H
P2xr4fl/fl-LysM-Cre- + P2X7-NB
P2xr4fl/fl-LysM-Cre+ + P2X7-NB
glycerol (mg/ml)
time (min)
I
fatty acids (mg/ml)
time (min)

Figure EV5. Related to Fig. 5. Myeloid P2X4 expression determines BAT degeneration.

(**A–G**) *P2rx4*$^{fl/fl}$-LysM^{Cre-} and *P2rx4*$^{fl/fl}$-LysM^{Cre+} mice were pretreated with the P2X7-inhibiting nanobody. Then, mice were daily injected with Eto and CL on two consecutive days. (**A**) BAT images of HE staining of individual mice. Scale bar, 50 µm. For better comparison, images of all individual mice are presented here, which includes the representative image shown in the main Fig. 5. (**B**) Quantification of lipid droplet area ($n = 5$, 3 images per section per mouse). (*$P = 0.0008$). (**C**) BAT images of MAC2 immunestaining of individual mice. Scale bar, 50 µm. For better comparison, images of all individual mice are presented here, which includes the representative image shown in the main Fig. 5. (**D**) Quantification of macrophages ($n = 5$, 3 images per section per mouse). (*$P = 0.0239$). (**E**) BAT images of Sirius Red staining of individual mice. Scale bar, 50 µm. For better comparison, images of all individual mice are presented here, which includes the representative image shown in the main Fig. 5. (**F**) Quantification of Sirius Red staining ($n = 5$, 3 images per section per mouse). (*$P = 0.0358$). (**G**) BAT images of UCP1 immunestaining of individual mice. Scale bar, 50 µm. For better comparison, images of all individual mice are presented here, which includes the representative image shown in the main Fig. 5. (**H**) + I *P2rx4*$^{fl/fl}$-LysM^{Cre-} and *P2rx4*$^{fl/fl}$-LysM^{Cre+} mice were pretreated with the P2X7-inhibiting nanobody. Then, mice were injected with CL. (**H**) Plasma glycerol levels at different time points after CL injection ($n = 6$). (**I**) Plasma fatty acid levels at different time points after CL injection ($n = 6$). Data are presented as mean values ± SEM. *$P < 0.05$ by Student's *t* test. *N* values indicate biological replicates.

 © The Author(s)

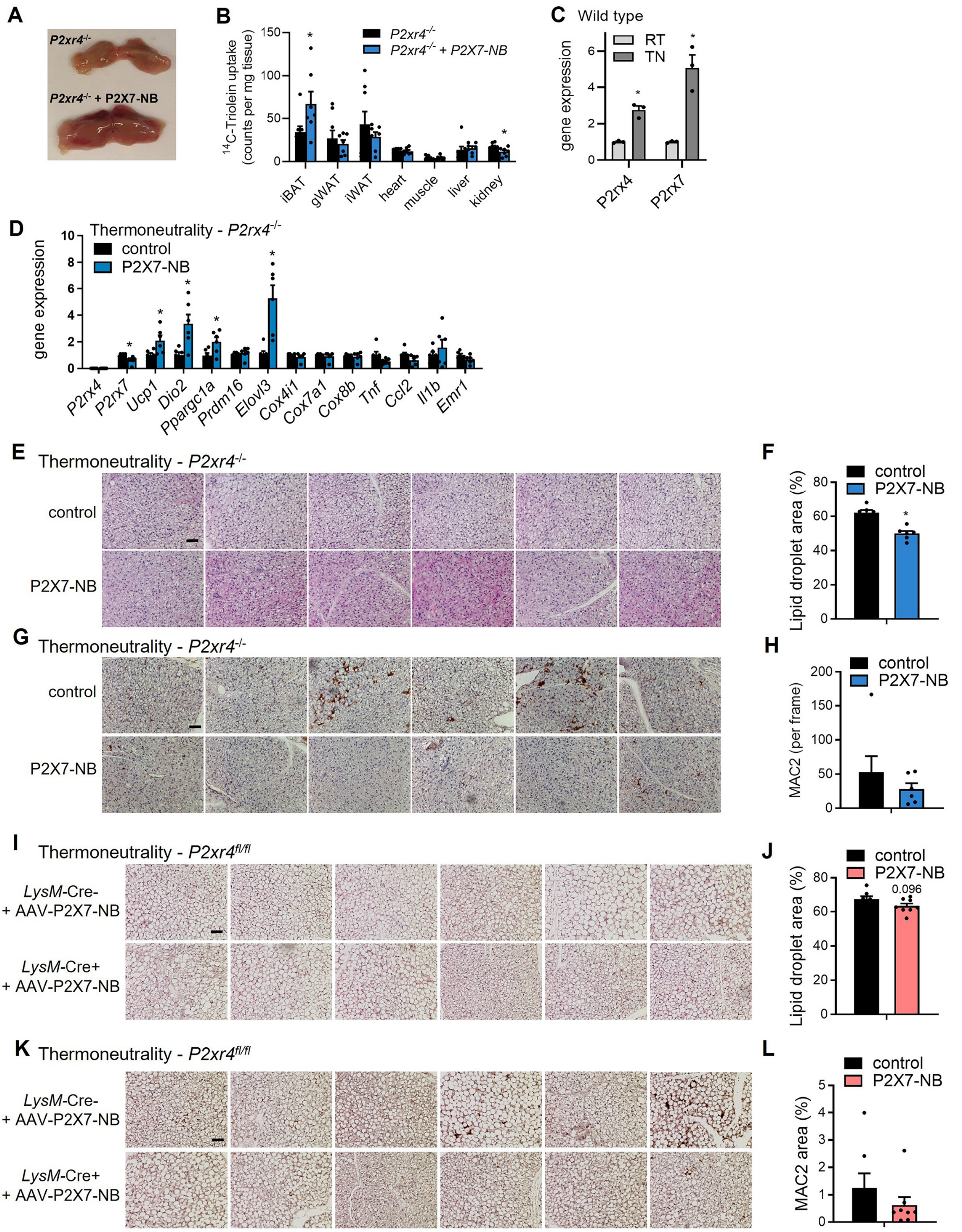
A
P2rx4-/-
P2rx4-/- + P2X7-NB
B
P2rx4-/-
P2rx4-/- + P2X7-NB
14C-Triolein uptake (counts per mg tissue)
iBAT gWAT iWAT heart muscle liver kidney
C Wild type
RT
TN
gene expression
P2rx4 P2rx7
D Thermoneutrality - P2rx4-/-
control
P2X7-NB
gene expression
P2rx4 P2rx7 Ucp1 Dio2 Ppargc1a Prdm16 Elovl3 Cox4i1 Cox7a1 Cox8b Tnf Ccl2 Il1b Emr1
E Thermoneutrality - P2rx4-/-
control
P2X7-NB
F
control
P2X7-NB
Lipid droplet area (%)
G Thermoneutrality - P2rx4-/-
control
P2X7-NB
H
control
P2X7-NB
MAC2 (per frame)
I Thermoneutrality - P2rx4fl/fl
LysM-Cre- + AAV-P2X7-NB
LysM-Cre+ + AAV-P2X7-NB
J
control
P2X7-NB
0.096
Lipid droplet area (%)
K Thermoneutrality - P2rx4fl/fl
LysM-Cre- + AAV-P2X7-NB
LysM-Cre+ + AAV-P2X7-NB
L
control
P2X7-NB
MAC2 area (%)

Figure EV6. Related to Fig. 6. P2X4/P2X7 blockade improves systemic glucose and lipid metabolism in distinct models of BAT degeneration.

(**A**, **B**) *P2rx4*$^{-/-}$ mice were pretreated with the P2X7-inhibiting nanobody or vehicle. Then, mice were daily injected with Eto and CL on two consecutive days. (**A**) Images of BAT after two days of Eto and CL treatment. (**B**) Uptake of i.v. injected TRL labeled with ^{14}C-triolein into various organs per mg tissue ($n = 7$–8). (*p = 0.0469; *p = 0.0265; left to right). (**C**) Gene expression of *P2rx4* and *P2rx7* in wild-type mice housed at room temperature (RT) or thermoneutrality (TN). (*P2rx4*: *p = 0.0015; *P2rx7*: *P = 0.0045; left to right). (**D–H**) *P2rx4*$^{-/-}$ mice receiving P2X7-inhibiting nanobody or vehicle (control) were housed at thermoneutrality (30 °C) for 2 weeks. (*P2rx7*: *P = 0.0068; *Ucp1*: *P = 0.0238; *Dio2*: *P = 0.0095; *Ppargc1a*: *P = 0.0263; *Elovl3*: *P = 0.0022; left to right). (**D**) Gene expression of purinergic receptors, thermogenic and inflammatory marker genes ($n = 6$). (**E**) BAT images of HE staining of individual mice. Scale bar, 50 µm. For better comparison, images of all individual mice are presented here, which includes the representative image shown in the main Fig. 6. (**F**) Quantification of lipid droplet area ($n = 6$, 3 images per section per mouse). (*P = 0.0003). (**G**) BAT images of MAC2 immunestaining of individual mice. Scale bar, 50 µm. For better comparison, images of all individual mice are presented here, which includes the representative image shown in the main Fig. 6. (**H**) Quantification of macrophages ($n = 6$, 3 images per section per mouse). (**I–L**) *P2rx4*$^{fl/fl}$-LysM^{Cre-} and *P2rx4*$^{fl/fl}$-LysM^{Cre+} mice received an AAV encoding for the P2X7-inhibiting nanobody (AAV-P2X7-NB) or control vector. Two weeks after infection, mice were housed at thermoneutrality (30 °C) for 2 weeks. (**I**) BAT images of HE staining of individual mice. Scale bar, 50 µm. For better comparison, images of all individual mice are presented here, which includes the representative image shown in the main Fig. 6. (**J**) Quantification of lipid droplet area ($n = 6$, 3 images per section per mouse). (**K**) BAT images of MAC2 immunestaining individual mice. Scale bar, 50 µm. For better comparison, images of all individual mice are presented here, which includes the representative image shown in the main Fig. 6. (**L**) Quantification of macrophages ($n = 6$, 3 images per section per mouse). Data are presented as mean values ± SEM. *$P < 0.05$ by Student's t test. N values indicate biological replicates.

 © The Author(s)
